# Fatal COVID-19 outcomes are associated with an antibody response targeting epitopes shared with endemic coronaviruses

**DOI:** 10.1172/jci.insight.156372

**Published:** 2022-07-08

**Authors:** Anna L. McNaughton, Robert S. Paton, Matthew Edmans, Jonathan Youngs, Judith Wellens, Prabhjeet Phalora, Alex Fyfe, Sandra Belij-Rammerstorfer, Jai S. Bolton, Jonathan Ball, George W. Carnell, Wanwisa Dejnirattisai, Christina Dold, David W. Eyre, Philip Hopkins, Alison Howarth, Kreepa Kooblall, Hannah Klim, Susannah Leaver, Lian Ni Lee, César López-Camacho, Sheila F. Lumley, Derek C. Macallan, Alexander J. Mentzer, Nicholas M. Provine, Jeremy Ratcliff, Jose Slon-Compos, Donal Skelly, Lucas Stolle, Piyada Supasa, Nigel Temperton, Chris Walker, Beibei Wang, Duncan Wyncoll, Oxford Protective T Cell Immunology for COVID-19 (OPTIC) consortium, Scottish National Blood Transfusion Service (SNBTS) consortium, Peter Simmonds, Teresa Lambe, John Kenneth Baillie, Malcolm G. Semple, Peter J.M. Openshaw, International Severe Acute Respiratory and emerging Infection Consortium Coronavirus Clinical Characterisation Consortium (ISARIC4C) investigators, Uri Obolski, Marc Turner, Miles Carroll, Juthathip Mongkolsapaya, Gavin Screaton, Stephen H. Kennedy, Lisa Jarvis, Eleanor Barnes, Susanna Dunachie, José Lourenço, Philippa C. Matthews, Tihana Bicanic, Paul Klenerman, Sunetra Gupta, Craig P. Thompson

**Affiliations:** 1 Peter Medawar Building for Pathogen Research,; 2 Nuffield Department of Medicine, and; 3 Department of Zoology, University of Oxford, Oxford, United Kingdom.; 4 Institute of Infection & Immunity, St George’s University of London, London, United Kingdom.; 5 Translational Gastroenterology Unit, Experimental Medicine Division, Nuffield Department of Medicine, John Radcliffe Hospital, Oxford, United Kingdom.; 6 Translational Research for Gastrointestinal Diseases, University Hospitals Leuven, Leuven, Belgium.; 7 The Jenner Institute Laboratories, University of Oxford, Oxford, United Kingdom.; 8 General Intensive Care service, St George’s University Hospital National Health Service (NHS) Trust, London, United Kingdom.; 9 Department of Veterinary Medicine, University of Cambridge, Cambridge, United Kingdom.; 10 Wellcome Centre for Human Genetics, Nuffield Department of Medicine,; 11 Oxford Vaccine Group, Department of Paediatrics, and; 12 Nuffield Department of Population Health, University of Oxford, Oxford, United Kingdom.; 13 Centre for Human & Applied Physiological Sciences, School of Basic & Medical Biosciences, Faculty of Life Sciences & Medicine, King’s College, London, United Kingdom.; 14 Department of Microbiology/Infectious Diseases, Oxford University Hospitals NHS Foundation Trust, John Radcliffe Hospital, Oxford, United Kingdom.; 15 Oxford Centre for Diabetes, Endocrinology and Metabolism, Churchill Hospital, and; 16 Future of Humanity Institute, Department of Philosophy, and; 17 Nuffield Department of Clinical Neurosciences, University of Oxford, Oxford, United Kingdom.; 18 Oxford University Hospitals NHS Foundation Trust, Oxford, United Kingdom.; 19 Department of Biochemistry, University of Oxford, Oxford, United Kingdom.; 20 Viral Pseudotype Unit, Medway School of Pharmacy, University of Kent, Chatham, United Kingdom.; 21 Meso Scale Diagnostics, Rockville, Maryland, USA.; 22 Intensive Care Medicine, Guy’s and St Thomas’ Hospital NHS Foundation Trust, London, United Kingdom.; 23 OPTIC consortium is detailed in Supplemental Acknowledgments.; 24 SNBTS consortium is detailed in Supplemental Acknowledgments.; 25 Roslin Institute, University of Edinburgh, Edinburgh, United Kingdom.; 26 NIHR Health Protection Research Unit in Emerging and Zoonotic Infections, Institute of Infection, Veterinary and Ecological Sciences, Faculty of Health and Life Sciences, University of Liverpool, Liverpool, United Kingdom.; 27 National Heart and Lung Institute, Imperial College, London, United Kingdom.; 28 ISARIC4C investigators are detailed in Supplemental Acknowledgments.; 29 School of Public Health, Faculty of Medicine, and; 30 Porter School of the Environment and Earth Sciences, Faculty of Exact Sciences, Tel-Aviv University, Tel-Aviv, Israel.; 31 National Microbiology Reference Unit, Scottish National Blood Transfusion Service, Edinburgh, United Kingdom.; 32 National Infection Service, Public Health England (PHE), Salisbury, United Kingdom.; 33 Siriraj Center of Research for Excellence in Dengue & Emerging Pathogens, Faculty of Medicine Siriraj Hospital, Mahidol University, Thailand.; 34 Chinese Academy of Medical Science (CAMS) Oxford Institute (COI), University of Oxford, Oxford, United Kingdom.; 35 Nuffield Department of Women’s & Reproductive Health, University of Oxford, John Radcliffe Hospital, Oxford, United Kingdom.; 36 Centre for Tropical Medicine and Global Health, Nuffield Department of Medicine, University of Oxford, Oxford, United Kingdom.; 37 Division of Biomedical Sciences, Warwick Medical School, University of Warwick, Coventry, United Kingdom.

**Keywords:** Immunology, Infectious disease, Adaptive immunity, Imprinting

## Abstract

The role of immune responses to previously seen endemic coronavirus epitopes in severe acute respiratory coronavirus 2 (SARS-CoV-2) infection and disease progression has not yet been determined. Here, we show that a key characteristic of fatal outcomes with coronavirus disease 2019 (COVID-19) is that the immune response to the SARS-CoV-2 spike protein is enriched for antibodies directed against epitopes shared with endemic beta-coronaviruses and has a lower proportion of antibodies targeting the more protective variable regions of the spike. The magnitude of antibody responses to the SARS-CoV-2 full-length spike protein, its domains and subunits, and the SARS-CoV-2 nucleocapsid also correlated strongly with responses to the endemic beta-coronavirus spike proteins in individuals admitted to an intensive care unit (ICU) with fatal COVID-19 outcomes, but not in individuals with nonfatal outcomes. This correlation was found to be due to the antibody response directed at the S2 subunit of the SARS-CoV-2 spike protein, which has the highest degree of conservation between the beta-coronavirus spike proteins. Intriguingly, antibody responses to the less cross-reactive SARS-CoV-2 nucleocapsid were not significantly different in individuals who were admitted to an ICU with fatal and nonfatal outcomes, suggesting an antibody profile in individuals with fatal outcomes consistent with an “original antigenic sin” type response.

## Introduction

Four human coronaviruses (HCoVs) are currently considered endemic. These include 2 beta-coronaviruses, HCoV-OC43 and HCoV-HKU1, as well as 2 alpha-coronaviruses, HCoV-229E and HCoV-NL63. Infection by these viruses causes a mild respiratory illness in the majority of people (1). Over the past 2 decades, 2 further beta-coronaviruses have also emerged, SARS-CoV-1 and MERS-CoV. Although both viruses have been more pathogenic than endemic coronaviruses, their transmission and subsequent spread have remained limited (2). In 2019 a fifth beta-coronavirus, SARS-CoV-2, emerged, which has led to a pandemic with over 100 million cases and upwards of 3 million deaths confirmed to date (3). Several studies have shown that prior infection with other HCoVs induces both cross-reactive antibody and T cell responses to SARS-CoV-2 (4–7). However, the response to shared epitopes and their relationship to disease progression have not been defined (8).

The spike protein of SARS-CoV-2, which is the primary vaccine target, consists of the S1 and S2 subunits (9, 10). The S1 subunit contains a variable receptor-binding domain (RBD), which mediates viral entry during the infection process via interaction with the angiotensin-converting enzyme 2 (ACE2) receptor. Antibodies targeting the RBD can be neutralizing and have been shown to correlate with protection (11). Endemic HCoV-induced antibody responses do not appear to cross-react with the SARS-CoV-2 RBD (9, 12) or at least do so infrequently (10, 13, 14). In contrast, several studies report that antibodies induced by prior HCoV infections cross-react with the SARS-CoV-2 S2 subunit (7–10), which is more conserved between beta-HCoV viruses. Several recent studies have shown that prior immunity induced by endemic beta-coronavirus infection to conserved epitopes of the S2 subunit inversely correlates with antibody response to novel parts of SARS-CoV-2 spike protein, such as the RBD (15, 16).

The targeting of previously seen parts of SARS-CoV-2 in preference to more novel parts of the virus occurs via a mechanism known as “original antigenic sin” (OAS), first described in 1960 by Thomas Francis. OAS can be defined as an immune response where a response to previously seen epitopes dominates the response to cognate antigens, when encountered at a later exposure (17).

Exposure to antigens shared between SARS-CoV-2 and related HCoVs may affect immunity and infection outcomes as a consequence of OAS (17). For OAS to manifest, antigens need to be shared between primary and secondary exposures (e.g., shared epitopes between HCoVs and SARS-CoV-2). Due to the development of memory, reexposure to any of the antigens present in the first exposure will result in a robust memory response that will overwhelm and potentially block the development of immune responses to new antigens associated with the secondary exposure (18). If immunity targeting the novel antigens present in the second exposure is needed for protection, OAS could affect disease progression and will differ across populations based on antigenic exposure histories.

Cross-reactive T cell responses have also been reported to be present in many individuals (5, 7, 19, 20), which may have been induced by prior infections with endemic HCoVs. These studies found CD4^+^ and CD8^+^ T cells reactive to SARS-CoV-2 spike peptide pools in blood samples from individuals unexposed to SARS-CoV-2 (5, 7, 20). This indicates that prior exposure to endemic HCoVs could confer a protective cross-reactive T cell response in a subset of the population (21).

In this study, we determine if antibody responses to shared endemic coronaviruses and SARS-CoV-2 epitopes could be used to characterize groups or cohorts with defined clinical outcomes. Consequently, we retrospectively tested samples against a panel of coronavirus antigens from individuals who previously had quantitative real-time PCR–confirmed asymptomatic infection, as well as individuals admitted to an intensive care unit (ICU) with severe COVID-19, half of whom died (22).

We also analyzed 2 large cohorts with SARS-CoV-2 neutralizing antibodies obtained from UK seroprevalence studies: one containing sera from blood donors and the other sera collected from pregnant women sampled at less than 14 weeks gestation (23, 24). These 2 cohorts did not have a precise clinical definition of SARS-CoV-2 infection severity. As a third control cohort, we included SARS-CoV-2–seronegative individuals from the same blood donor seroprevalence study (24). Further details of the cohorts can be found in Table 1 and Supplemental Figures 6 and 7; supplemental material available online with this article; https://doi.org/10.1172/jci.insight.156372DS1

We show that in comparison with individuals admitted to an ICU with nonfatal COVID-19 outcomes, those with fatal outcomes had a lower antibody response to the SARS-CoV-2 RBD and N-terminal domain of the spike protein. However, there was no significant difference in antibody responses to the S2 domain of the spike protein, which is more conserved among endemic beta-coronaviruses. Individuals with fatal COVID-19 outcomes also showed no difference in antibody responses to the less cross-reactive nucleocapsid (25). To our knowledge this is the first time that the response to shared endemic beta-coronavirus and SARS-CoV-2 epitopes has been shown to be a marker of fatal COVID-19 outcomes.

## Results

### Individuals with fatal COVID-19 outcomes have lower antibody responses to the SARS-CoV-2 spike protein but not the SARS-CoV-2 nucleocapsid.

**Figure 1:**
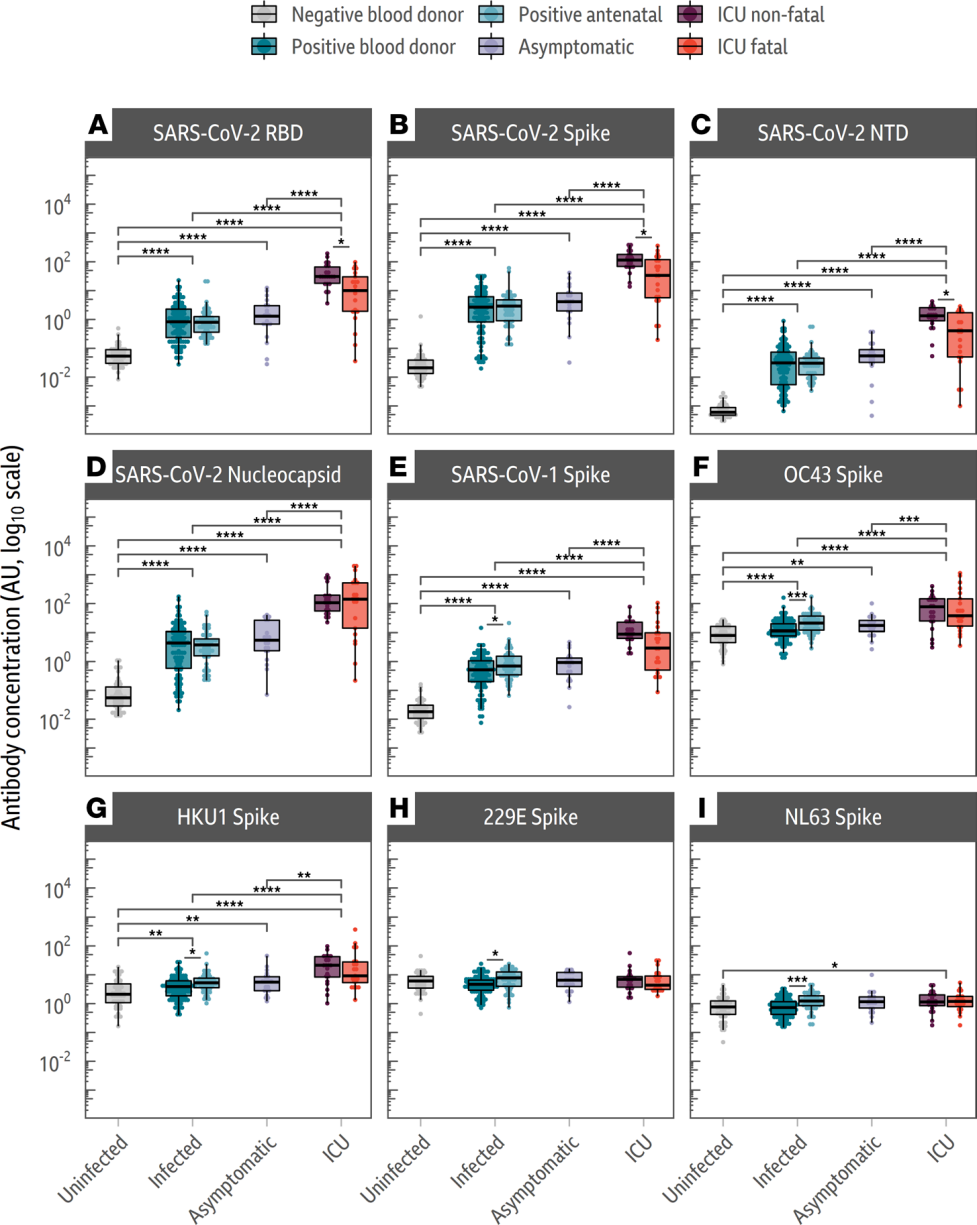
Individuals admitted to ICUs with fatal COVID-19 outcomes have lower responses to the SARS-CoV-2 spike protein but not the SARS-CoV-2 nucleocapsid. Box plots comparing antibody concentrations for (**A**–**D**) SARS-CoV-2 spike domains and nucleocapsid, (**E**) SARS-CoV-1 full-length spike, and (**F**–**I**) HCoV full-length spike antigens. For all box plots, plots depict the minimum and maximum values (whiskers), and the shaded box indicates the upper and lower quartiles and the median. Sample groups (background uninfected, infected, asymptomatic, and ICU with fatal and nonfatal outcomes) are given on the *x* axis. Subgroups are denoted by color. The average response to all SARS-CoV-2 antigens was elevated in individuals admitted to an ICU with COVID-19, and no differences were observed between the infected and asymptomatic groups. Individuals admitted to an ICU with fatal COVID-19 outcomes had a lower response to SARS-CoV-2 RBD (*t* test: *P* = 0.01), spike (*t* test: *P* = 0.02), and NTD (*t* test: *P* = 0.02) than individuals admitted to an ICU with nonfatal COVID-19 outcomes. The data in this figure were generated using the MSD V-PLEX assay. *t* tests were used to assess significance, and the reported *P* values were adjusted for multiple comparisons using the Holm-Bonferroni method. *P < 0.05, **P < 0.01, ***P < 0.001, ****P < 0.0001.

We used a multi-spot assay system (Meso Scale Diagnostics [MSD] V-PLEX) to quantify total antibody responses to the SARS-CoV-2 nucleocapsid, the SARS-CoV-2 RBD, N-terminal domain (NTD) of the spike, the full-length spike, as well as the spike proteins of the 4 HCoVs and SARS-CoV-1 (Figure 1) (26). In-house indirect enzyme-linked immunosorbent assays (ELISAs) were also developed for the SARS-CoV-2 RBD and full-length spike, in addition to the full-length alpha- and beta-HCoV spike proteins, to confirm the results produced by the MSD V-PLEX assay via a second independent method. Both the MSD assay and in-house ELISA results correlated well (Supplemental Figure 1). A schematic of the proteins and their subunits and domains used in this analysis can be found in Supplemental Figure 2, while the variability of the associated proteins is shown in Supplemental Figures 3–5.

We found that the antibody titers to the SARS-CoV-2 spike and nucleocapsid were low in convalescent sera from individuals with asymptomatic infections and substantially higher in individuals admitted to ICU with severe COVID-19 outcomes (sampled during acute infection) (Figure 1). However, among those admitted to ICU with severe COVID-19, individuals with fatal outcomes consistently exhibited lower titers to SARS-CoV-2 spike antigens than those with nonfatal outcomes; responses to the SARS-CoV-2 RBD (Figure 1A, *t* test: *P* = 0.01), and NTD (Figure 1C, *t* test: *P* = 0.02), as well as the full-length spike (*t* test: *P* = 0.02), were all higher in the nonfatal cases. In contrast, no significant difference in antibody responses to the second SARS-CoV-2 antigen, the nucleocapsid protein, was identified (Figure 1D, *t* test: *P* = 0.99).

### Beta-coronavirus responses are enriched in individuals with fatal COVID-19 outcomes.

We next compared responses to endemic HCoV spike antigens in the cohorts to determine how they correlated with COVID-19 clinical outcome. All cohorts previously infected with SARS-CoV-2 showed increased responses to the endemic beta-HCoV spike proteins relative to the unexposed background cohort (Figure 1), suggesting that infection with SARS-CoV-2 induces increased cross-reactive beta-HCoV responses, as reported elsewhere (10, 12, 15).

We found that the increased reactivity to the beta-HCoV spike proteins was also broadly associated with COVID-19 severity. The response to the HCoV-OC43 spike antigen was significantly larger for individuals admitted to an ICU with COVID-19 than either the infected (Figure 1F, *t* test: *P* = 2.93 × 10^–6^) or asymptomatic groups (Figure 1F, *t* test: *P* = 3.73 × 10^–4^). Similar increases were observed for the beta-HCoV HKU1 spike protein (Figure 1G), although these were smaller in magnitude in comparison with those associated with the HCoV-OC43 spike protein. Increases in responses to shared SARS-CoV-2/HCoV epitopes, termed “back boosts” by several papers, have been previously observed during natural infection and after vaccination (15, 27). Among individuals admitted to an ICU with severe COVID-19, antibody responses to endemic beta-HCoV spike proteins were not statistically different between those with fatal and nonfatal outcomes, unlike responses to the SARS-CoV-2 spike protein (Figure 1B, *t* test: *P* = 0.83).

There was no comparative increase in antibody responses to alpha-HCoV (HCoV-NL63 and HCoV-229E, Figure 1, H and I) spike proteins following SARS-CoV-2 infection in either the blood donor or asymptomatic cohorts. However, smaller increases in responses to alpha-HCoV spike protein were detected in the ICU fatal/nonfatal outcome groups as well as the antenatal control group. For all endemic HCoVs, the antenatal cohort had an elevated antibody spike protein response in comparison with the blood donor cohort, which could not be explained by biases in age or sex, but we postulate these trends could be due to environmental differences (Figure 1, H and I).

**Figure 2:**
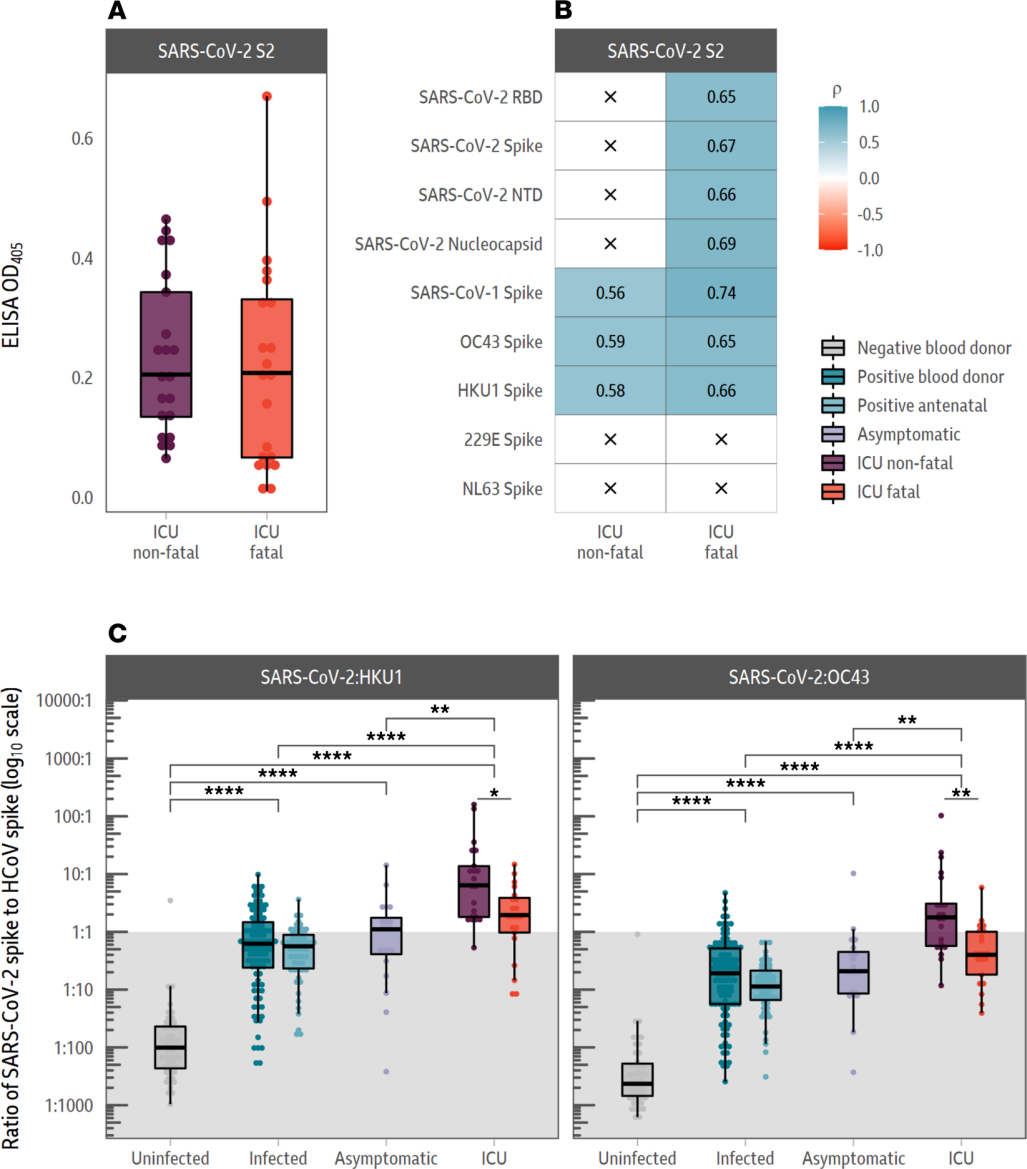
Individuals with fatal COVID-19 outcomes have immune responses enriched in antibodies targeting shared SARS-CoV-2 and endemic beta-coronavirus epitopes. (**A**) Antibody responses to the S2 subunit of the SARS-CoV-2 spike protein are not statistically different in individuals admitted to an ICU with fatal or nonfatal COVID-19 outcomes. (**B**) S2 antibody responses correlate with the SARS-CoV-2 responses in individuals admitted to an ICU with fatal COVID-19 outcomes but not nonfatal outcomes. An *x* indicates the absence of a correlation. (**C**) Ratio of beta-HCoV (HCoV-HKU1 or HCoV-OC43) spike response to SARS-CoV-2 spike response. The gray division in the figure indicates the point at which the ratio of SARS-CoV-2 spike response to beta-HCoV response is lower than 1. The *t* test was used to assess significance, and the reported *P* values were adjusted for multiple comparisons using the Holm-Bonferroni method, in **A** and **C**. Spearman’s correlations are shown for each pair of antigens in **B**. *P < 0.05, **P < 0.01, ****P < 0.0001.

The S2 subunit of the SARS-CoV-2 spike protein is conserved to a greater extent between beta-HCoVs than other more variable parts of the spike, such as the RBD or NTD (ref. 15; Supplemental Figure 2). As responses to the SARS-CoV-2 spike protein but not the beta-HCoV spike proteins were reduced in individuals with fatal COVID-19 outcomes relative to those with nonfatal outcomes, we next analyzed responses to the S2 subunit of the SARS-CoV-2 spike (Figure 2, A and B).

To test responses to the S2 subunit of the SARS-CoV-2 spike protein, we developed an S2 subunit indirect ELISA. In contrast to the other SARS-CoV-2 spike antigens (the RBD, the NTD of the spike, as well as the full-length spike) measured by both the MSD V-PLEX assay and in-house ELISA, there was no difference in magnitude of the SARS-CoV-2 S2 ELISA responses between the fatal and nonfatal cohorts (Figure 2A: *t* test: *P* = 0.99).

Furthermore, we found that individuals admitted to an ICU with fatal outcomes had antibody responses to the SARS-CoV-2 RBD, NTD, full-length spike, and nucleocapsid that correlated strongly with the SARS-CoV-2 S2 antibody responses (Figure 2B). These correlations were absent in individuals in an ICU nonfatal COVID-19 outcomes group and are denoted by a black *x*.

The ratio of total antibody response to the beta-coronavirus spike was then determined as a proportion of the SARS-CoV-2 response (Figure 2C). This demonstrated that the antibody response to SARS-CoV-2 in individuals with fatal COVID-19 outcomes was enriched for antibodies that bind both SARS-CoV-2 and endemic beta-HCoV spike proteins.

### ACE2-binding inhibition and pseudotyped SARS-CoV-2 microneutralization assay responses correlate with disease severity.

**Figure 3:**
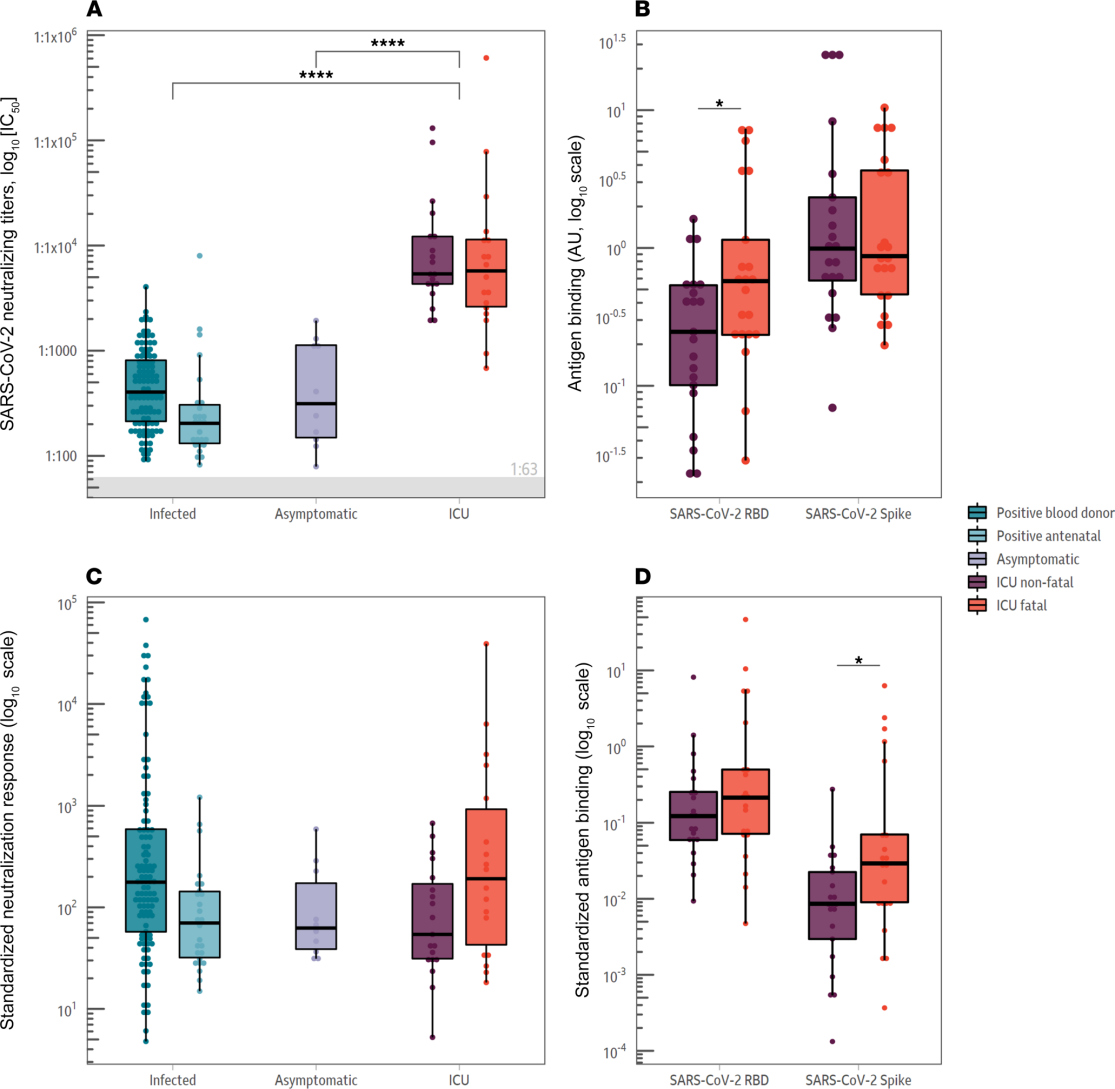
Neutralizing antibody levels correlate with disease severity. (**A**) Neutralizing antibody levels. Neutralization titers were higher in the individuals admitted to the ICU with COVID-19. There was no significant difference between individuals admitted to an ICU with fatal or nonfatal COVID-19 outcomes (*t* test: *P* = 0.99). (**B**) ACE2 inhibition assay results. Samples were also analyzed with an MSD R-PLEX ACE2 inhibition assay. The level of ACE2-binding inhibition was not statistically significant for the full-length spike protein, but the individuals admitted to an ICU with fatal COVID-19 outcomes showed statistically lower ACE2-RBD binding inhibition in comparison with the nonfatal ICU cohort (*t* test: *P* = 0.02). (**C**) Neutralizing antibody levels as a proportion of total spike antibody response. There was no statistically significant difference between any of the groups. (**D**) ACE2-binding inhibition as a proportion of total spike antibody response. ACE2-binding inhibition responses were significantly lower in individuals with fatal COVID-19 outcomes in comparison with those with nonfatal COVID-19 outcomes when measured by the R-PLEX full-length spike but not the RBD inhibition assays (*t* test: RBD; *P* = 0.25, spike; *P* = 0.018). *t* tests were used to assess significance, and the reported *P* values were adjusted for multiple comparisons using the Holm-Bonferroni method. *P < 0.05, ****P < 0.0001.

The neutralizing antibody response has been shown to be a key correlate of protection against SARS-CoV-2 infection (11, 28, 29). Three assays were run to determine neutralizing antibody responses: a pseudotyped SARS-CoV-2 microneutralization assay as well as 2 R-PLEX competition assays measuring the binding capacity of ACE2 to the SARS-CoV-2 spike and RBD, respectively (Figure 3). The assays show entry inhibition, a widely used proxy for live virus SARS-CoV-2 neutralization capacity (24, 26, 30).

Neutralizing antibody titers were comparable in both the ICU groups with fatal and nonfatal COVID-19 outcomes, as measured using a pseudotyped SARS-CoV-2 microneutralization assay (Figure 3A, *t* test: *P* = 0.96). R-PLEX ACE2 competition assays using the full-length spike and RBD as antigens were also run. The R-PLEX assay showed no significant difference in binding inhibition between ACE2 and the spike protein (*t* test: *P* = 0.83). However, there was significantly lower inhibition of binding between the RBD and ACE2 in the MSD R-PLEX assay for individuals with fatal COVID-19 outcomes (Figure 3B; *t* test: *P* = 0.02).

Comparison of the ratio of neutralizing antibody responses to total SARS-CoV-2 spike antibody responses showed that ACE2 binding inhibition responses were significantly lower in individuals with fatal COVID-19 outcomes in comparison with those with nonfatal COVID-19 outcomes when measured by the MSD R-PLEX full-length spike but not the RBD inhibition assays (Figure 3D, *t* test: RBD *P* = 0.25, spike *P* = 0.018; ref. 13). Again, there was no difference between neutralizing responses in individuals admitted to an ICU with fatal COVID-19 outcomes and those with nonfatal outcomes when neutralization was measured by pseudotype neutralization assay (Figure 3C, *t* test: *P* = 0.26).

### The SARS-CoV-2 antibody response in individuals with fatal COVID-19 outcomes correlates with responses to endemic beta-coronavirus spike proteins.

**Figure 4:**
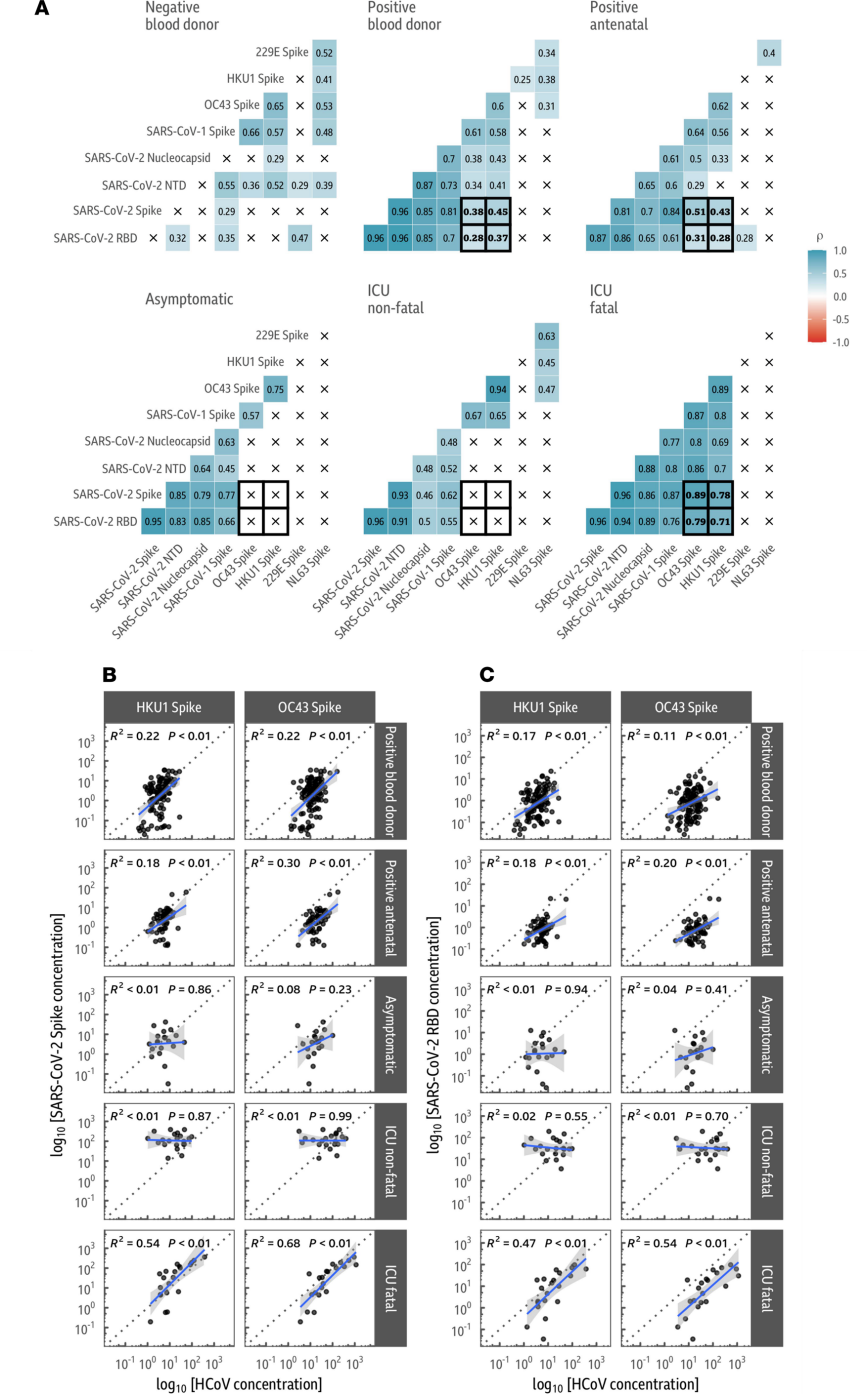
In fatal COVID-19 outcomes, antibody responses to SARS-CoV-2 are highly correlated with antibody responses to the endemic beta-coronavirus spike proteins. (**A**) Correlation between SARS-CoV-2 and endemic coronavirus responses. Spearman’s rank correlations (ρ) are shown for each pair of antigens, split by sample group. There is a positive correlation between all SARS-CoV-2 antigens in all cohorts exposed to SARS-CoV-2. Significant correlations are found between SARS-CoV-2 antigens and endemic beta-HCoVs (HCoV-OC43 and HCoV-HKU1) in the SARS-CoV-2 antibody-positive blood donor and antenatal groups as well as the ICU fatal outcome group. These correlations are absent in the asymptomatic and nonfatal outcome from severe COVID-19 groups. The correlation between endemic beta-HCoVs and SARS-CoV-2 antigens is considerably weaker in the larger positive blood donor and antenatal cohorts than in the ICU fatal outcome group. Responses to the SARS-CoV-2 spike (**B**) and RBD (**C**) correlate with beta-coronavirus spike responses in individuals with fatal COVID-19 outcomes. Correlations are shown with a linear model fit between the concentration of 2 SARS-CoV-2 antigens and the endemic viruses HCoV-OC43 and HCoV-HKU1. The best fit line is shown in blue with 95% confidence intervals in gray; the dotted gray division denotes a 1:1 response to both antigens. There is a strong positive association between SARS-CoV-2 spike/RBD and the endemic HCoVs in the fatal outcomes from severe COVID-19 group, which is absent in the similarly sized asymptomatic and nonfatal outcomes from severe COVID-19 groups.

We then analyzed how the SARS-CoV-2 antibody response correlated with the antibody response to beta-HCoV spike proteins. We calculated the Spearman’s rank correlation coefficients for all pairs of antigens from the MSD V-PLEX assay, split by cohort (Figure 4A). Notably, in the ICU fatal COVID-19 outcome group, responses to the SARS-CoV-2 spike were strongly correlated with the HCoV-OC43 and HCoV-HKU1 spike antigens (Spearman’s rank correlation: *P* = 0.89, *P* = 8 × 10^–8^ and *P* = 0.78, *P* = 4 × 10^–5^, respectively). This correlation was present not only for the full-length spike antigen but also for the NTD (Spearman’s rank correlation: HCoV-OC43 *P* = 5 × 10^–7^; HCoV-HKU1 *P* = 4 × 10^–4^) and RBD (Spearman’s rank correlation: HCoV-OC43 *P* = 2 × 10^–5^; HCoV-HKU1 *P* = 3 × 10^–4^) of the spike, as well as the SARS-CoV-2 nucleocapsid (Spearman’s rank correlation: HCoV-OC43 *P* = 5. × 10^–7^; HCoV-HKU1 *P* = 4 × 10^–4^; Figure 4). Notably, we could not identify statistically significant correlations in the similarly sized asymptomatic and ICU nonfatal COVID-19 outcome groups (Figure 4A).

A linear model fit on the log-scale was used to analyze the correlation of the magnitude of response to either the SARS-CoV-2 NTD or RBD of the spike, the full-length spike, and the SARS-CoV-2 nucleocapsid (Figure 4B) with the HCoV-HKU1 and HCoV-OC43 spike responses in the asymptomatic and ICU fatal/nonfatal COVID-19 outcome groups. Responses between SARS-CoV-2 antigens and the beta-HCoVs correlated strongly in the fatal COVID-19 outcome group with consistently high *R*^2^ values, indicating that for those with fatal COVID-19 outcomes, the SARS-CoV-2 de novo antibody response was strongly linked with the responses to shared SARS-CoV-2/HCoV spike protein epitopes. Importantly, these trends were consistent when the linear models were age adjusted (Supplemental Table 1).

Our larger blood donor and antenatal control cohorts also showed a weaker correlation between SARS-CoV-2 antibody response and endemic beta-HCoV spike antibody responses, indicating that this phenomenon can also be found to a lesser extent in the general population if sample size is substantially increased, as reported elsewhere (17).

### Preferentially targeted epitopes map to the HCoV-OC43 S2 subunit of the spike protein but not the HCoV-OC43 nucleocapsid.

**Figure 5:**
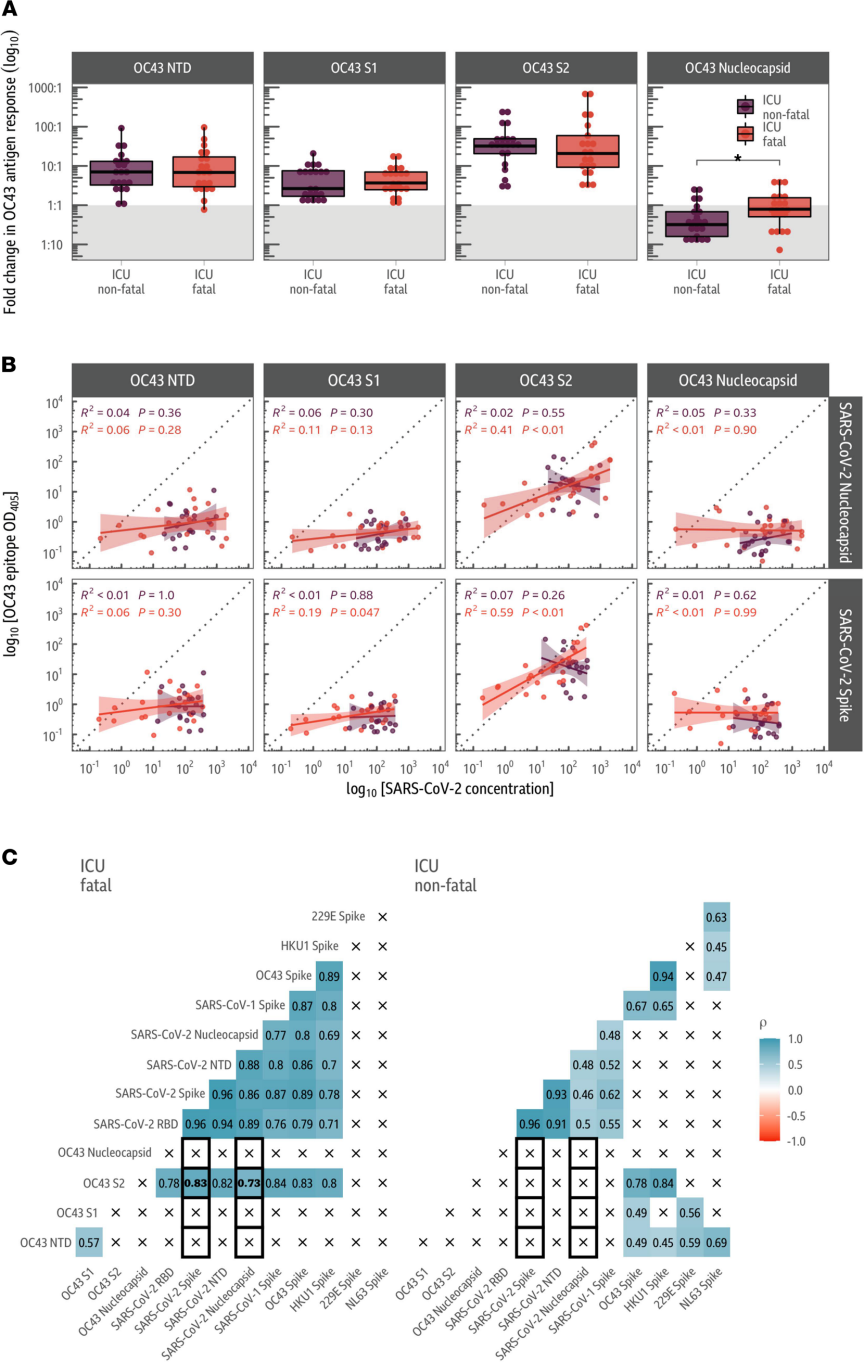
Antibody responses are directed against the S2 subunit of the HCoV-OC43 spike protein. (**A**) Fold change in responses to various domains/subunits in the HCoV-OC43 spike protein and nucleocapsid. Indirect ELISAs were used to analyze responses to the NTD, S1 subunit and S2 subunit of the HCoV-OC43 spike protein, in addition to the HCoV-OC43 nucleocapsid. Fold change via ELISA was determined relative to the average value in the SARS-CoV-2 antibody-negative blood donor cohort as indicated by the gray division in the figure. Antibody levels are increased against all antigens apart from the nucleocapsid, with the largest increase in antibody response to the S2 subunit of the spike protein. (**B**) Correlation in responses between SARS-CoV-2 antigens and HCoV-OC43 spike protein domains and nucleocapsid. The log-scale OD_405_ values from the HCoV-OC43 spike and nucleocapsid ELISAs (along the rows) is compared to the MSD V-PLEX SARS-CoV-2 results (columns). A linear model fit on the log-scale is annotated with the associated 95% confidence intervals and *R*^2^ and *P* values. Values and model fits for the nonfatal COVID-19 outcomes group are given in purple, while red is used for the fatal outcome group. The HCoV-OC43 S2 subunit ELISA result is only correlated with the concentration of SARS-CoV-2 antibodies in the fatal group. (**C**) Correlations between ELISA and MSD V-PLEX SARS-CoV-2 assay responses. Responses to the S2 subunit of HCoV-OC43 are strongly correlated with the MSD concentration of SARS-CoV-2 antibodies in those who died from COVID-19 but not those who survived. Notably, there is a positive correlation between the S2 subunit response and the HCoV-OC43 and HCoV-HKU1 spike responses in the fatal COVID-19 outcome group. *t* tests were used to assess significance, and the reported *P* values were adjusted for multiple comparisons using the Holm-Bonferroni method, in **A**. Spearman’s rank correlations (ρ) are shown for each pair of antigens in **B** and **C**.

In cases of COVID-19 with fatal outcomes, responses to shared epitopes in the HCoV-OC43 spike protein increased to a greater extent than those to the HCoV-HKU1 spike protein (Figure 1, F and G). Consequently, to identify the location of the beta-HCoV epitopes causing the correlation, we chose to subdivide the HCoV-OC43 spike protein into the NTD (amino acid [aa] 1–419) as well as the S1 (aa 1–794) and S2 (aa 766–1304) subunits (Figure 5A). The various domains and subunits of the HCoV-OC43 spike protein and the HCoV-OC43 nucleocapsid analyzed can be found in Supplemental Figure 8.

Responses to infection in both ICU fatal and nonfatal SARS-CoV-2 groups demonstrated an increase in response to all regions of the HCoV-OC43 spike protein analyzed, although the response to the S2 subunit was considerably greater, indicating that the majority of shared SARS-CoV-2 and beta-HCoV epitopes reside in the S2 subunit.

There were no significant differences in the fold change of responses to the NTD or S1 or S2 subunits of the HCoV-OC43 spike protein between the ICU nonfatal or fatal outcome groups (Figure 5A). There were median fold change increases of 6.93 (*t* test: *P* = 1 × 10^–3^), 2.48 (*t* test: *P* = 4 × 10^–3^), and 31.4 (*t* test: *P* = 2 × 10^–18^) to the NTD and S1 and S2 HCoV-OC43 spike domains, respectively, across the ICU fatal and nonfatal outcome groups in comparison with the blood donor negative control group.

We then fitted a linear regression between the log concentration of response (as measured by antibody titer) between the HCoV-OC43 responses (the NTD, the S1 and S2 subunits of the spike, as well as the nucleocapsid) and either the SARS-CoV-2 full-length spike protein or nucleocapsid (Figure 5B). In individuals with fatal COVID-19 outcomes, there was a strong correlation between antibody responses to the HCoV-OC43 S2 subunit and the SARS-CoV-2 spike (Spearman’s rank correlation: *P* = 6.48 × 10^–7^), which extended to the SARS-CoV-2 RBD (Spearman’s rank correlation: *P* = 3.62 × 10^–5^) and NTD of the spike protein (Spearman’s rank correlation: *P* = 3.38 × 10^–6^), as well as the SARS-CoV-2 nucleocapsid (Spearman’s rank correlation: Figure 5, B and C; *P* = 0.0018). The SARS-CoV-2 spike S2 subunit has previously been shown to be the major target of antibodies induced by prior endemic coronavirus infection (9, 10). In contrast to the S2 spike subunit, antibody responses to both the HCoV-OC43 S1 subunit and NTD correlated poorly with SARS-CoV-2 antibody response in individuals with fatal COVID-19 outcomes (Figure 5C). These trends were also consistent when the linear models were age adjusted (Supplemental Table 2).

We next looked in more detail at the HCoV-OC43 nucleocapsid response. Wratil et al. have recently shown that responses to the endemic coronavirus nucleocapsids (HKU1, OC43, 229E, and NL63) do not increase during COVID-19, and these responses can therefore be used as markers of immunity to endemic coronaviruses prior to SARS-CoV-2 infection (25).

To that end, we analyzed the HCoV-OC43 nucleocapsid response in our cohorts. In agreement with Wratil et al. and Aguilar-Bretones et al. (25, 31), we found that HCoV-OC43 nucleocapsid levels in both the ICU fatal and nonfatal COVID-19 outcome groups did not increase above background population levels. Background levels were determined by analysis of the control uninfected blood donor cohort (Figure 5A, indicated by the gray division). Furthermore, there was also no correlation between the HCoV-OC43 nucleocapsid levels and either the spike or the SARS-CoV-2 nucleocapsid (Figure 5B, Spearman’s rank correlation: *P* = 0.99 and *P* = 0.9, respectively).

When comparing between the ICU cohorts, we found that there was a significantly higher HCoV-OC43 nucleocapsid response in individuals with fatal COVID-19 outcomes in contrast to individuals with nonfatal outcomes (Figure 5A; *t* test: *P* = 4 × 10^−4^), indicating that there was likely to be greater, or more recent, exposure to HCoV-OC43 coronavirus prior to SARS-CoV-2 infection in individuals with fatal COVID-19 outcomes.

## Discussion

Our study shows that in fatal COVID-19 outcomes, the antibody response to the SARS-CoV-2 spike was enriched for antibodies that bind to conserved epitopes shared with endemic beta-coronaviruses. The majority of these epitopes are found within the S2 subunit of the SARS-CoV-2 spike (Figure 2C and Figure 5).

Individuals with fatal COVID-19 outcomes have a lower de novo antibody response to the SARS-CoV-2 RBD and NTD of the SARS-CoV-2 spike protein (Figure 1, A–C). These regions have been shown to be more divergent between the beta-HCoVs than the S2 subunit (9, 17). In contrast, the response to the more conserved SARS-CoV-2 S2 subunit of the spike was not significantly different between individuals admitted to an ICU, regardless of outcome (Figure 2, A and B).

Importantly, antibody responses to a second SARS-CoV-2 antigen, the nucleocapsid protein, were not significantly different between individuals admitted to an ICU with fatal and nonfatal COVID-19 outcomes (Figure 1D). As has been reported in Wratil et al. and Aguilar-Bretones et al. (25, 31), we found that unlike the HCoV-OC43 spike protein, antibody responses to the HCoV-OC43 nucleocapsid did not increase upon SARS-CoV-2 infection (Figure 5A). However, OC43 nucleocapsid antibody responses were higher in individuals admitted to an ICU with fatal COVID-19 outcomes, compared with individuals with nonfatal outcomes. This could indicate that individuals admitted to an ICU with fatal outcomes had higher levels of immunity to HCoV-OC43 prior to SARS-CoV-2 infection than individuals admitted to an ICU with nonfatal outcomes (Figure 5A; ref. 26).

These observations are compatible with the OAS concept, whereby prior immune responses compromise de novo responses to a related antigen (25). In this case, prior immunity to the endemic beta-coronavirus HCoV-OC43 or HCoV-HKU1 epitopes shared with SARS-CoV-2 could impair the immune response to novel SARS-CoV-2 epitopes (17, 18).

In this context, upon infection with SARS-CoV-2, memory B cells produced during an individual’s previous exposure to either HCoV-OC43 or HCoV-HKU1 recognize conserved epitopes in SARS-CoV-2 and would outcompete naive B cells, targeting novel parts of the spike protein, in the germinal center reaction. This could, in theory, lead to diminishment of an effective antibody response, if less protective regions, such as the S2 region of the SARS-CoV-2 spike, are targeted by memory B cells (31, 32).

Several studies have shown that antibodies targeting the RBD of SARS-CoV-2 are associated with protection (11, 29). In addition to these studies, Dejnirattisai et al. found that antibodies that bound the S2 subunit were less potent neutralizers than antibodies that bound the RBD in focus reduction neutralization tests (32, 33).

Therefore, the inhibition or reduction of RBD antibody responses could provide a mechanism by which OAS may lead to a worse clinical outcome. Similar phenomena have been observed for influenza viruses and for SARS-CoV-2 but have not yet been associated with clinical outcome to our knowledge (15, 34, 35).

It is unlikely that immunosenescence is responsible for the observations in this study as responses in the fatal and nonfatal groups to the second SARS-CoV-2 antigen, the nucleocapsid, were not significantly different (Figure 1D, *t* test; *P* = 0.99). If de novo responses were generally impaired in the fatal COVID-19 outcome group, then responses to both SARS-CoV-2 antigens should be equally impaired.

However, it is important to note that although we describe an association, our data do not show direct correlation or provide irrefutable evidence for an immunological mechanism. These findings suggest a number of potential hypotheses, which need validating in further cohorts.

Alternatively, the results outlined in this study could also be due to a yet-to-be-defined malfunctioning immune response whereby the immune response to certain novel antigens is inhibited. We cannot also exclude the possibility that some non-RBD binding antibodies could be disease enhancing.

Finally, individuals with severe COVID-19 admitted to an ICU had much stronger immune responses to both the spike and nucleocapsid than individuals with asymptomatic infection. This is partially due to the timing of the sampling of the asymptomatic individuals in question (during the convalescent phase) but also due to the greater disease burden and length of infection imposed by severe infection, leading to a greater antibody response, which may make the underlying mechanisms easier to differentiate (29). We would expect that the levels of antibodies in individuals with severe COVID-19 would eventually drop over time to levels similar to those in our convalescent asymptomatic samples (36).

Our data are also in agreement with other studies such as that of Atyeo et al., which outlines that there is a greater spike response in individuals surviving severe COVID-19 in contrast to individuals who die as a consequence of their infections (Figure 1, A–C; ref. 37). The antibody response in individuals with fatal COVID-19 outcomes is skewed toward nucleocapsid targeting (37). Consequently, our data agree with Atyeo et al. that the spike/nucleocapsid ratio can be used as a measure of disease severity. Within our cohorts, the ratio of spike/nucleocapsid response was skewed toward the nucleocapsid in individuals with fatal COVID-19 outcomes, in contrast with those with nonfatal outcomes (Figure 1, B and D).

Our study also builds on Atyeo et al. by demonstrating that the response targeted against the spike can be divided into a cross-reactive S2 response, which did not differ between ICU fatal and nonfatal COVID-19 outcome groups (Figure 2), and a de novo response targeting the RBD and NTD of the spike, which caused the difference in spike response between the 2 outcomes (Figure 1, A–C). Furthermore, our data also agree with those of Aguilar-Bretones et al., which showed IgG B cell clones, activated by prior coronavirus infection, are boosted to the greatest extent in individuals with severe COVID-19 (31).

However, there exist several limitations of our study. The time frame of sampling has been suggested to be critical in the ability to identify differences in the responses among severely ill COVID-19 patients (31). The single time point sampled in this study limits the window in which the appearance of de novo responses can be examined in fatal COVID-19 cases (Supplemental Figure 9). Consequently, an earlier time point might indicate that neutralizing antibodies are generally lower, as opposed to only 1 out of 3 neutralization or ACE2-binding assays showing this feature (Figure 2). This would match with the consistently lower IgG RBD, NTD, and spike antibody responses measured by ELISA (Figure 3). The quality of the neutralizing antibody response may be affected by OAS-induced blocking of antibody responses to new antigens expressed by SARS-CoV-2 absent in beta-coronaviruses (10, 21).

We also note that our S2 ELISA was run in the prefusion form and that further work needs to be undertaken regarding the inhibition of fusions and other mechanisms, such as antibody-dependent cell cytotoxicity, with regard to the role of S2 SARS-CoV-2 spike binding antibodies.

The study is further limited by the absence of longitudinal samples. Longitudinal samples would be ideal to determine the level of prior immunity to endemic HCoVs. In addition to this, whether the key contributing factors are age, sex, time since the last beta-coronavirus infection, infection with specific strains, or just serendipity is beyond the scope of this study. Individuals in this study were likely to have been infected with early SARS-CoV-2 variants, and it is also intriguing to consider how these trends would manifest in those infected with more recent emergent variants. This may have implications impacting vaccine efficacy, and to this end, the same phenomena should be studied in those vaccinated with spike proteins from early variants of SARS-CoV-2, then exposed to new variants or updated vaccines.

## Methods

### ELISA.

SARS-CoV-2 spike and RBD as well as HCoV-229E, HCoV-NL63, HCoV-HKU1, and HCoV-OC43 spike IgG antibody responses were measured using in-house indirect ELISAs. Further work to characterize the location of the conserved epitopes between HCoV-OC43 and SARS-CoV-2 used the HCoV-OC43, NTD, and S1 and S2 subunits. Spike and RBD proteins were produced as per Amanat et al. (38). Further information regarding the antigens used in the analysis can be found in Supplemental Table 3.

Nunc-Immuno 96-well plates (Thermo Fisher Scientific) were coated with 1.0 μg/mL of antigen in PBS and left overnight at 4°C. Plates were washed 3 times with 0.1% PBS–Tween (PBS/T), then blocked with casein in PBS for 1 hour at room temperature (RT). Serum or plasma was diluted in casein–PBS solution at dilutions ranging from 1:50 to 1:20,000 before being added to Nunc-Immuno 96-well plates in triplicate. Plates were incubated for 2 hours before being washed 6 times with PBS/T. Secondary antibody rabbit anti-human whole IgG conjugated to alkaline phosphatase (catalog A3187-1ML, MilliporeSigma) was added at a dilution of 1:1000 in casein–PBS solution and incubated for 1 hour at RT. After a final wash, plates were developed by adding 4-nitrophenyl phosphate substrate in diethanolamine buffer (Pierce), and OD was read at 405 nm using an ELx800 microplate reader (Cole Parmer).

The positive reference standard was used on each plate to produce a standard curve. A monoclonal antibody standard was used for the RBD/spike ELISAs (35). Pooled HCoV highly reactive sera were used as a standard for the HCoV spike ELISAs.

### MSD V-PLEX assay.

IgG antibody responses to SARS-CoV-2 spike, RBD, NTD, and nucleocapsid and the spike proteins of SARS-CoV-1, HCoV-229E, HCoV-NL63, HCoV-HKU1, and HCoV-OC43 were assessed using the MSD Multi-Spot Assay System. Precoated plates (Coronavirus panel 2) were incubated at RT with Blocker A solution for at least 30 minutes while being shaken at 500–700 rpm. Serum or plasma was diluted 1:500 to 1:50,000, and samples were added to the plates in duplicate. Plates were incubated for 2 hours at RT, while being shaken at 500–700 rpm throughout. A 1× working concentration of the SULFO-TAG anti-human IgG Detection Antibody was prepared (R32AJ-5, MSD). After incubation with the samples, the plates were washed 3 times with 1× MSD wash buffer. Prepared detection antibody solution was added to the plates, which were incubated at RT for 1 hour, while being shaken. Plates were then washed 3 times with 1× MSD Wash buffer. To read the assay results, MSD GOLD Read Buffer B (provided ready to use) was added to the plate. The plates were read on a QuickPlex SQ 120 (MSD) immediately after adding the buffer.

A 7-point calibration curve of the standards was prepared, along with a negative control. An additional 3 positive controls provided with the kit were also run on every plate. All standards and controls were run in duplicate. Data from the assay were analyzed using MSD Discovery Workbench software, which averaged all the duplicates, and generated and fitted all the data to standard curves (26).

Some of the 10 MSD assay plates (enough for 350 samples) were gifted by MSD, in addition to the loan for the QuickPlex SQ 120 assay system. No agreements were made regarding publication or promotion of the system.

### MSD ACE2 competition assay.

The ability of antibodies present in serum/plasma to inhibit the binding of ACE2 to the SARS-CoV full-length spike proteins and RBD domains was assessed using the COVID-19 ACE2 competition assay (MSD). The assay can be used to estimate the neutralizing activity of the antibodies present in the samples.

Precoated plates were incubated at RT with MSD blocker A solution for at least 30 minutes while being shaken at 500–700 rpm. Serum or plasma was diluted at 1:10 to 1:100, and samples were added to the plates in duplicate. Plates were incubated for 1 hour at RT, while being shaken at 500–700 rpm throughout. A 1× working concentration of the SULFO-TAG ACE2 detect was prepared. After incubation with the samples, the plates were washed 3 times with 1× MSD Wash buffer. Prepared SULFO-TAG ACE2 solution was added to the plates, which were incubated at RT for a further 1 hour, while being shaken. Plates were then washed 3 times with 1× MSD Wash buffer. To read the assay results, MSD GOLD Read Buffer B (provided ready to use) was added to the plate. Plates were read immediately after adding the buffer on a QuickPlex SQ 120 (MSD).

A 7-point calibration curve of the standards was prepared along with a negative control. All standards were run in duplicate. Data from the assay were analyzed using MSD Discovery Workbench software, which averaged all the duplicates, and generated and fitted all the data to standard curves.

### Pseudotyped virus microneutralization assay.

A lentivirus-based pseudotyped virus system was used to display the SARS-CoV-2 spike protein on its surface using a synthetic codon optimized SARS-CoV-2 expression construct (National Center for Biotechnology Information [NCBI] reference sequence: YP_009724390.1). Pseudotyped viruses were generated by transfecting HEK293 T/17 cells (ATCC) with 1.0 μg of codon optimized spike protein (plasmid pcDNA3.1), 1.0 μg of gag/pol (plasmid p8.91), and 1.5 μg of a luciferase reporter construct (plasmid pCSFLW) as part of a plasmid-OptiMEM/PEI solution. Transfections were performed in 10 mL of media, DMEM 10% FCS, 1% penicillin-streptomycin, and 20% l-glutamate, and left for 24 hours at 37°C 5% CO_2_. Fresh media were added to the cells before leaving them at 37°C 5% CO_2_ for 48 hours. Supernatant was then harvested and stored at –80°C.

Target cells (HEK293T) were transfected using Fugene (Promega) 24 hours prior to assay setup with 2.75 μg of ACE2 expression plasmid and 250 ng of TMPRSS2 expression plasmid (GeneArt, Thermo Fisher Scientific) (24).

### Phylogenetic analysis.

Consensus sequences were generated by aligning sequences using MUSCLE and curated in AliView before generating consensus sequences using Bioconductor R package ConsensusSequence. Amino acid sequences were aligned using MAFFT (https://mafft.cbrc.jp/alignment/software/). Maximum likelihood trees were generated using MEGA X (39) with 1000 bootstrap replicates. Trees were midpoint rooted and bootstrap support of greater than 70% is indicated. Trees were visualized using Figtree (http://tree.bio.ed.ac.uk/software/figtree/).

### Variability analysis.

A total of 5000 sequences were randomly selected from the GISAID SARS-CoV-2 protein database. These were curated so that only whole sequences were included. All HCoV-HKU1 and HCoV-OC43 sequences were downloaded from the NCBI. The proteins sequences were aligned using MUSCLE before being curated. The sequences were aligned to either the SARS-CoV-2 spike or nucleocapsid sequences. Only sequences containing full-length sequences with run of uncalled bases less than 3 were used. The variability determined by counting the possible mismatches at each amino acid position was then determined via counting the number of different amino acids.

### Statistics.

Over dispersed variables were transformed onto the logarithmic scale (base 10) for between group comparisons for V-PLEX platform concentrations, ELISA OD and neutralizing titers. Unless otherwise specified, a 2-tailed *t* test assuming unequal variances was used to test for differences in the mean responses; values were analyzed on the logarithmic (base 10) scale unless otherwise stated. A Holm-Bonferroni correction was applied to *P* values for multiple comparisons. In cases where a fold change or ratio is calculated, the log-scale group means can be compared to 0 using a 2-tailed *t* test to determine if the group differs from equal concentrations of antigens. Data pertaining to difference in means were tested for normality using the Shapiro-Wilk test. We did not find violations of normality and hence used a Welch 2-tailed *t* test for such differences. Reported correlations are Spearman’s rank, as the measure is nonparametric and robust to transformation. *P* values less than or equal to 0.05 were considered statistically significant.

### Study approval.

Ethical approval was obtained for the SNBTS anonymous archive — IRAS project number 18005. SNBTS blood donors gave fully informed consent to virological testing, donation was made under the SNBTS Blood Establishment Authorisation, and the study was approved by the SNBTS Research and Sample Governance Committee IRAS project number 18005.

The ISARIC WHO Clinical Characterisation Protocol-UK (CCP-UK) protocol was developed by international consensus in 2012–2014 and activated in response to the SARS-CoV-2 pandemic on January 17, 2020. This is an actively recruiting prospective cohort study recruiting across the United Kingdom (40). Study materials, including protocol, revision history, case report forms, study information, and consent forms, are available online (https://isaric4c.net/protocols/). Ethical approval was given by the South Central — Oxford C Research Ethics Committee in England (Ref: 13/SC/0149) and by the Scotland A Research Ethics Committee (Ref: 20/SS/0028).

The antenatal samples were collected during routine antenatal care appointments in the Oxfordshire area. Samples were taken during the first trimester of pregnancy (generally between 8 and 12 weeks’ gestation) between April 14 and June 15, 2020. Ethical approval was obtained from the South Central — Oxford C Research Ethics Committee (Ref: 08/H0606/139).

Patients and health care workers comprising the asymptomatic cohort were recruited from the John Radcliffe Hospital in Oxford, United Kingdom, between March and May 2020. Patients identified during the SARS-CoV-2 pandemic were screened and recruited into the Sepsis Immunomics (IRAS260007) and ISARIC WHO CCP-UK (IRAS126600). Patients were sampled at least 28 days from their positive PCR test. The ICU patients were enrolled as part of an ongoing prospective observational study, AspiFlu (ISRCTN Registry ISRCTN51287266), at St George’s Hospital, London, United Kingdom. Ethical approval for AspiFlu was given by the Wales Research Ethics Committee, ref: 19/WA/0310. Researchers working with the samples in the laboratory were blinded to the clinical outcomes of the ICU patients during testing. None of the study participants received convalescent plasma therapy.

## Author contributions

Conceptualization was done by PK and CPT; methodology was developed by ALM, RSP, ME, PP, NT, CW, SBR, TL, and KK; software programming was done by RSP, JSB, UO, and JL; formal analysis was done by RSP, CPT, and KK; investigation was done by ALM, ME, AF, JW, HK, JSB, JB, JR, LL, and LS; resources were provided by SBR, GC, WD, CD, DWE, AH, SL, CLC, SFL, DM, AJM, NMP, DS, P Supasa, NT, CW, BW, DW, KK, PH, JSC, JM, MGS, JKB, PJMO, ISARIC4C investigators, OPTIC consortium, SNBTS consortium, P Simmonds, MGS, TL, JKB, MT, MC, GS, SHK, LJ, EB, SD, PCM, and TB; data curation management was done by ALM, RSP, ME, JY, JW, SBR, GC, WD, CD, DWE, AH, SL, CLC, SFL, DM, AJM, PH, JSC, JM, NMP, DS, P Supasa, NT, CW, BW, DW, ISARIC4C investigators, OPTIC consortium, and SNBTS consortium; original draft preparation was done by ALM, RSP, and CPT; all authors reviewed and approved the final manuscript; visualization preparation was done by RSP, UO, JL, and CPT; supervision was performed by TL, SD, PK, and CPT; project administration was done by ALM and CPT; and funding acquisition was done by PK, SG, CPT, MGS, JKB, and PJMO.

## Supplementary Material

Supplemental data

## Figures and Tables

**Table 1:**
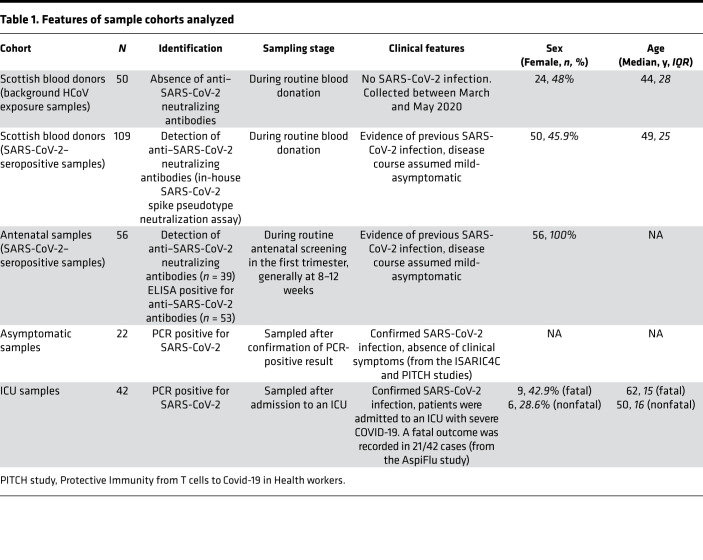
Features of sample cohorts analyzed

## Version 1 05/24/2022

In-Press Preview

## Version 2 07/08/2022

Electronic publication
