# Supplementary material for: Fatal COVID-19 outcomes are associated with an antibody response targeting epitopes shared with endemic coronaviruses
Source: JCI Insight. 2022 Jul 8;7(13):e156372. doi: 10.1172/jci.insight.156372 (PMC9310533; doi:10.1172/jci.insight.156372)
Supplement: Supplemental data [file jciinsight-7-156372-s149.pdf]

Supplementary Material

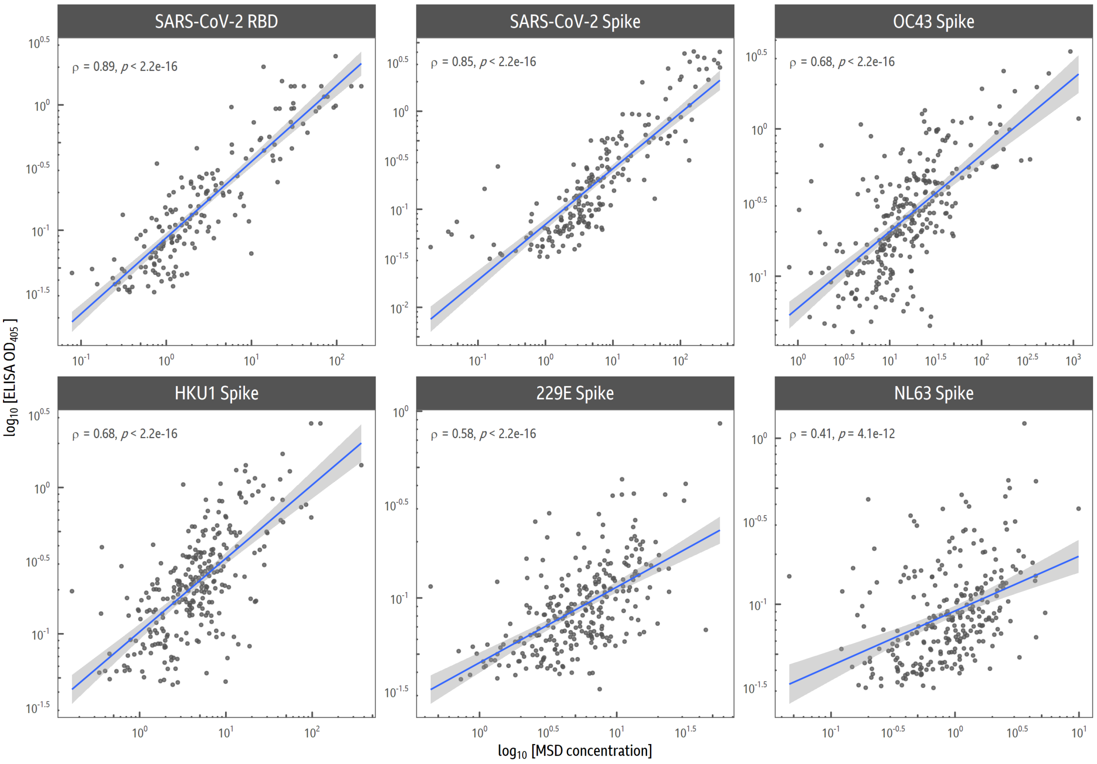

Figure S1: Correlation of MSD VPLEX and ELISA data. The correlation of results across the two assays for the SARS-CoV-2 full-length spike and RBD domains, along with endemic coronavirus spike antigens is shown with Spearman’s Rank correlations.

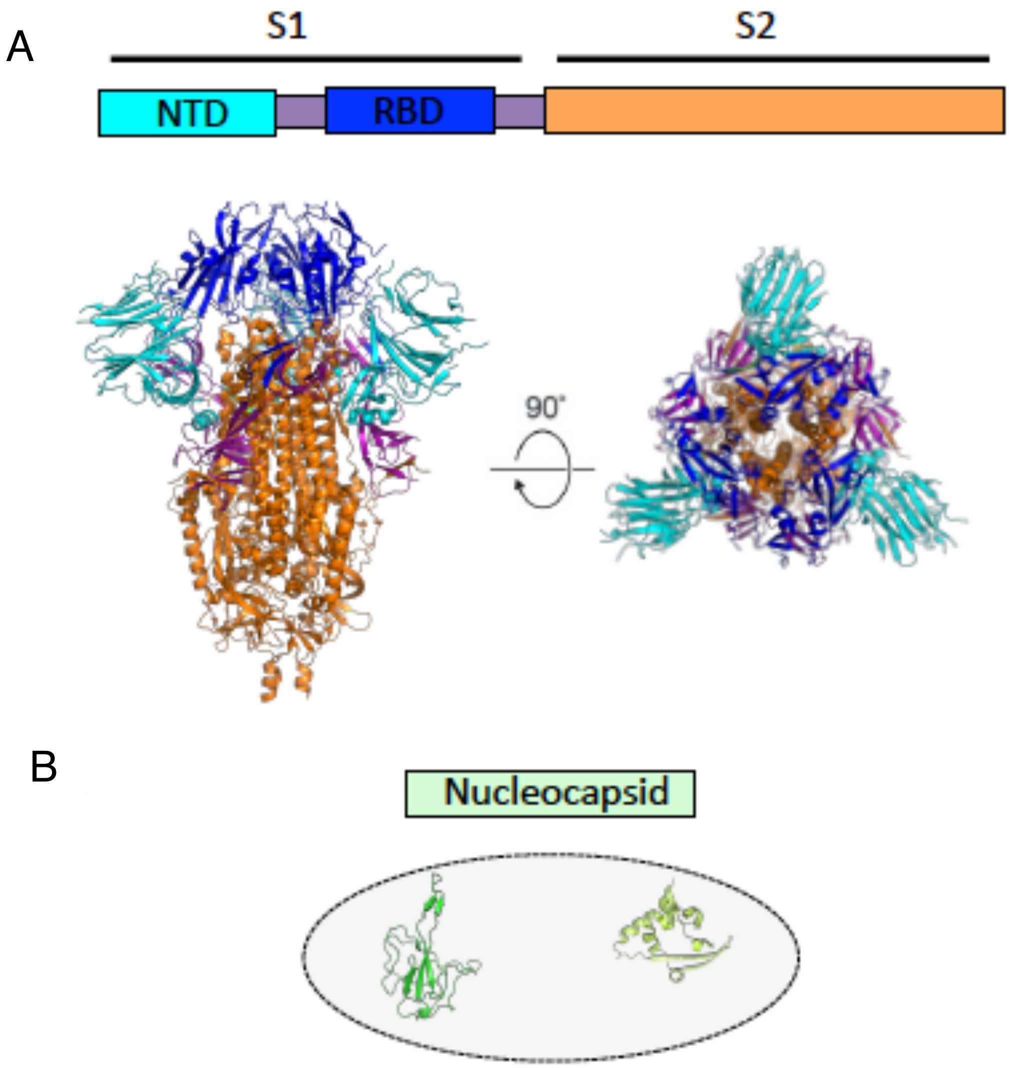

Figure S2. A schematic of the subunits and domains of the SARS-CoV-2 spike protein and SARS-CoV-2 nucleocapsid used in the MSD V-PLEX assay and ELISAs. (A) Various regions and subunits of the spike were analysed. Variable regions such as the N-terminal (NTD) and receptor-binding domain (RBD) in the S1 subunit of the spike protein, in addition to the more conserved S2 subunit were analysed.. **(B) A second antigen was also assessed, the SARS-CoV-2 nucleocapsid, which was not subdivided into domains.** A full crystal structure exists for the SARS-CoV-2 spike, whilst only partial structures of the N-terminal and RNA binding domain regions of the SARS-CoV-2 nucleocapsid exist. These are shown above.

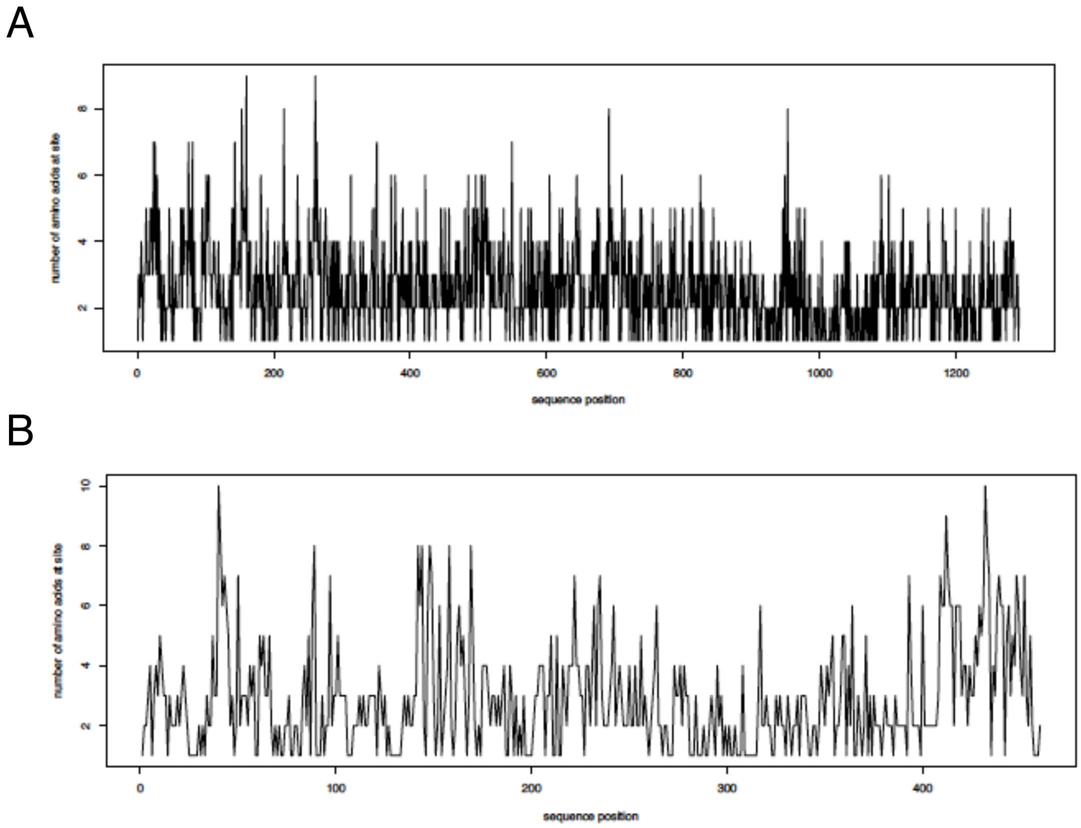

Figure S3. Variability of aligned beta-HCoV spike and nucleocapsid proteins. (**A) Variability analysis of spike protein**: 3,653 SARS-CoV-2, 100 HCoV-HKU1 and 100 HCoV-OC43 spike sequences. (**B) Variability analysis of nucleocapsid protein:** 4,845 SARS-CoV-2, 100 HCoV-HKU1 and 100 HCoV-OC43 nucleocapsid sequences. Further information regarding the analysis can be found in the Methods section.

Spike proteins

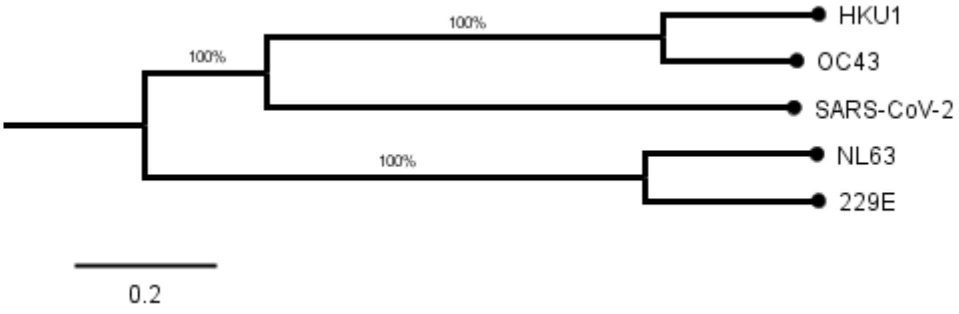

Nucleocapsid proteins

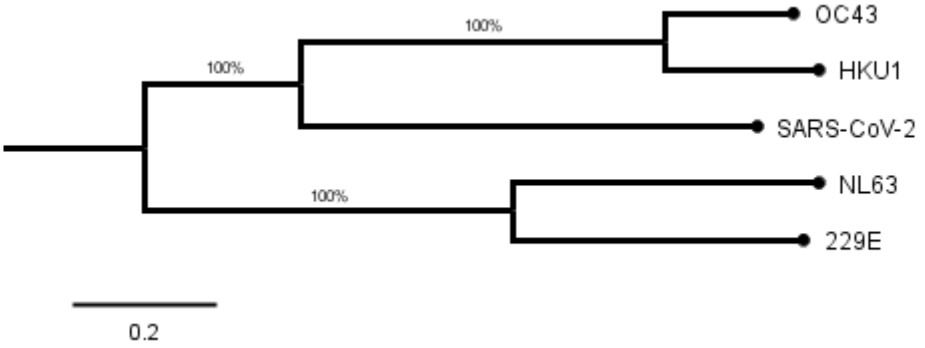

Figure S4: Phylogenetic analysis of coronavirus spike and nucleocapsid protein consensus sequences.

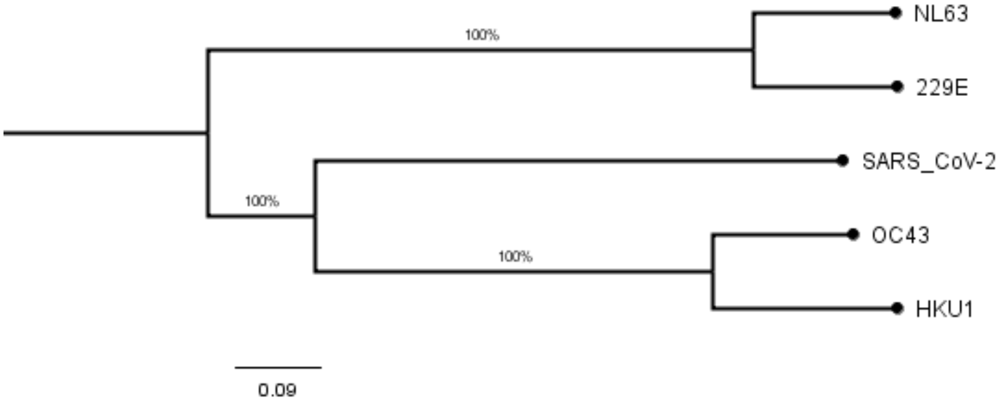

Figure S5: Phylogenetic analysis of coronavirus spike S2 subunit consensus sequences.

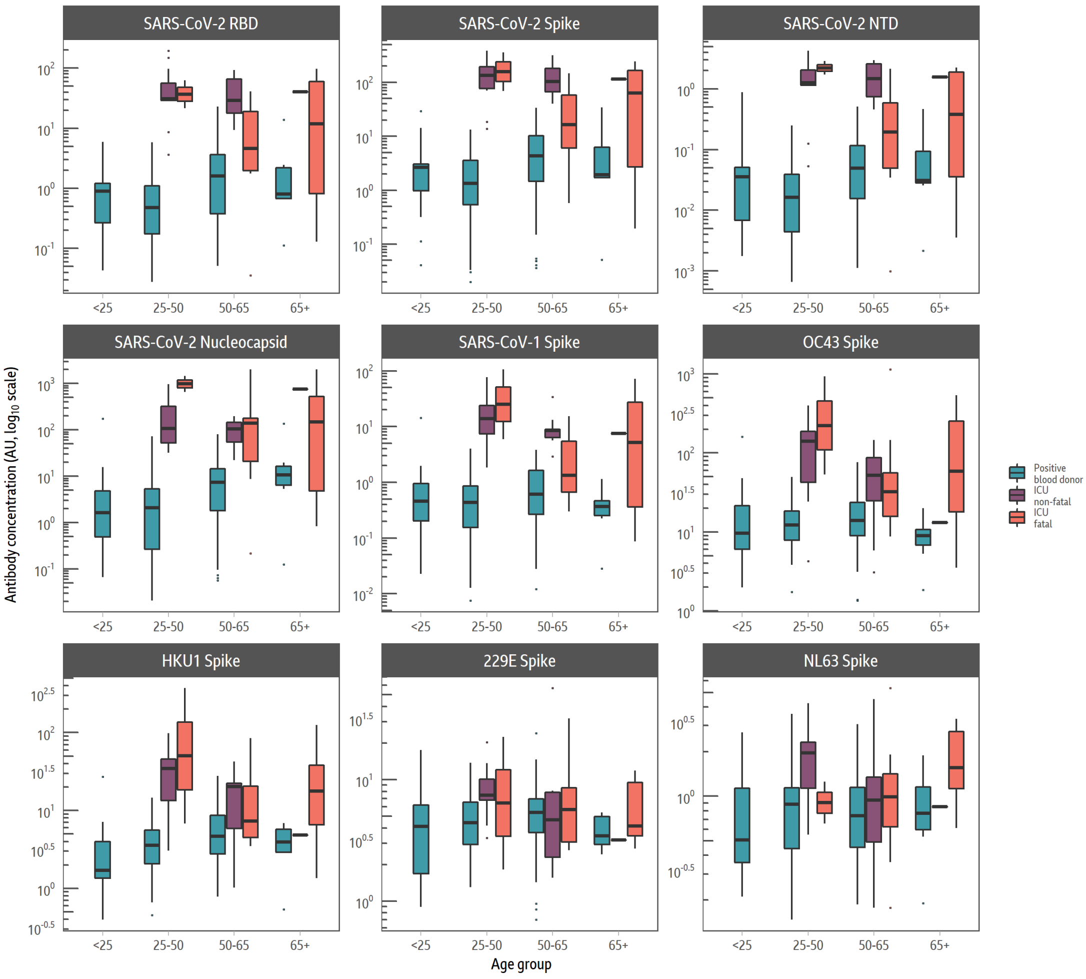

Figure S6: IgG antibody responses categorised by age in the different groups.

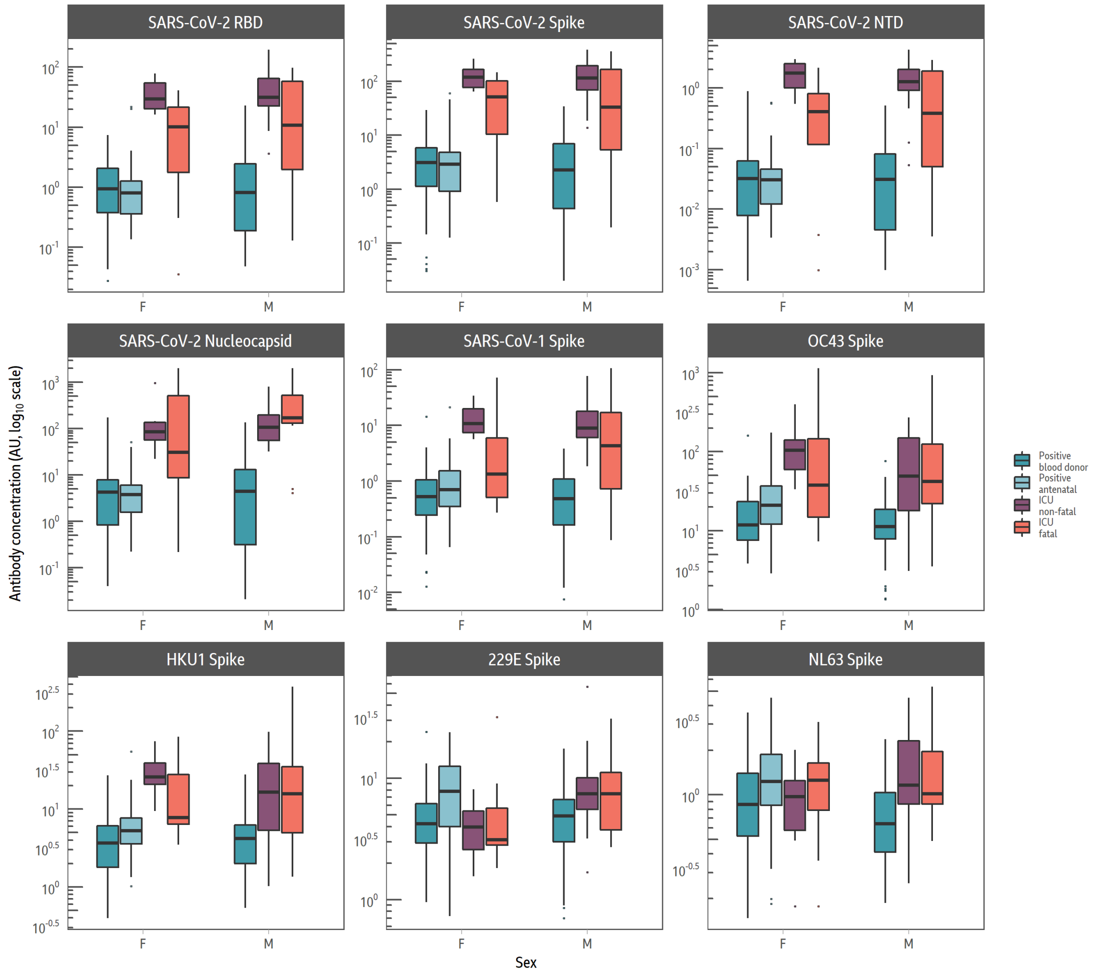

Figure S7: IgG antibody responses categorised by sex in the different groups.

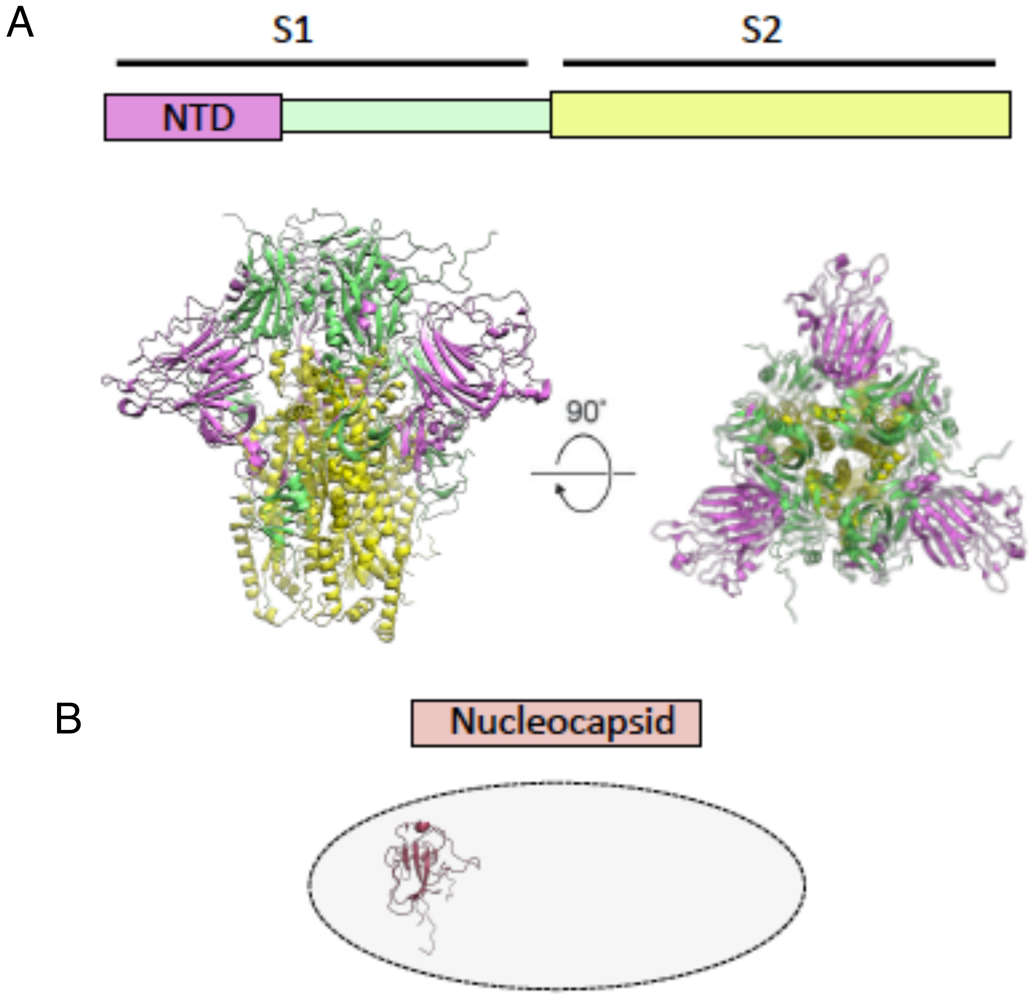

Figure S8. A schematic of the subunits and domains of the HCoV-OC43 spike protein and HCoV-OC43 nucleocapsid used in ELISAs. **(A) Various domains and subunits of the HCoV-OC43 spike were analysed.** Variable regions such as the N-terminal (NTD) in the S1 subunit of the spike protein in addition to the more conserved S2 subunit were analysed. **(B) A second antigen, the HCoV-OC43 nucleocapsid, was analysed which was not subdivided into domains.** A full crystal structure exists for the HCoV-OC43 spike, whilst only a partial structure of the N-terminal domain exists.

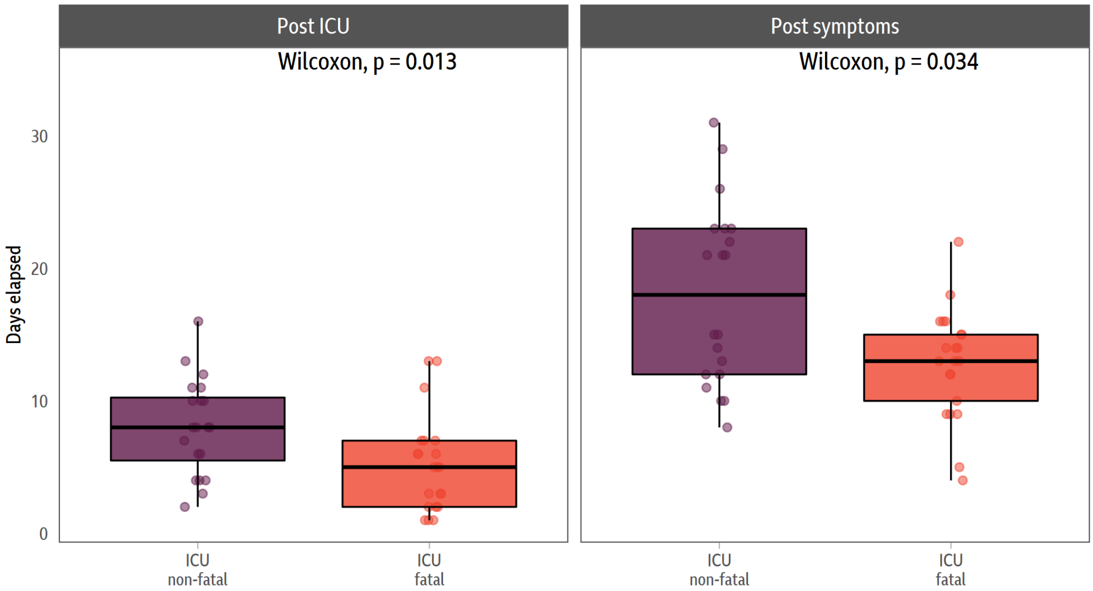

Figure S9: Days from post-symptomatic and entry to ICU in the fatal and non-fatal groups. In both instances there is statistically significant differences.

Antigens	Adjusted R-squared for the fatal ICU cohort	P-value for the fatal ICU cohort	Adjusted R-squared for the non-fatal ICU cohort	P-value for the non-fatal ICU cohort
OC43 spike & SARS-CoV-2 Spike	0.66	6*10-6	0.04	0.81
HKU spike & SARS-CoV-2 Spike	0.597	0.0002	0.05	0.69
OC43 spike & SARS-CoV-2 RBD	0.496	0.0002	0.07	0.42
HKU spike & SARS-CoV-2 RBD	0.42	0.001	0.1	0.296

Table S1. Adjusted R-squared, and p-values for the slope of the antigen in age-adjusted linear models based on those in Figure 4.

Antigens	Adjusted R-squared for the fatal ICU cohort	P-value for the fatal ICU cohort	Adjusted R-squared for the non-fatal ICU cohort	P-value for the non-fatal ICU cohort
OC43 S2 &SARS-CoV-2 Spike	0.66	1.5*10-5	0.13	0.16
OC43 S2 &SARS-CoV-2 Nucleocapsid	0.396	0.0011	0.07	0.37
OC43 Nucleocapsid & SARS-CoV-2 Nucleocapsid	-0.03	0.865	-0.06	0.35
OC43 Nucleocapsid & SARS-CoV-2 Spike	-0.02	0.77	-0.09	0.61
OC43 S1 & SARS-CoV-2 Spike	0.179	0.099	-0.02	0.99
OC43 S1 & SARS-CoV-2 Nucleocapsid	0.11	0.25	0.015	0.38
OC43 NTD & SARS-CoV-2 Nucleocapsid	-0.04	0.28	0.13	0.51
OC43 NTD & SARS-CoV-2 Nucleocapsid	-0.04	0.3	0.11	0.77

Table S2. Adjusted R-squared, and p-values for the slope of the antigen in age-adjusted linear models based on those in Figure 5.

Supplementary Materials

Methods Table 1: Protein sequences used in ELISAs

**Protein**	**Expressed in**	**Provider**	**Reference**
HCoV-HKU1 spike	Insect cells	Sino Biological	
HCoV-OC43 spike	Insect cells	Sino Biological	
HCoV-NL63 spike	Insect cells	Sino Biological	
HCoV-229E spike	Insect cells	Sino Biological	
SARS-CoV-2 spike	HEK 293T	Produced in-house	Amanat *et al.* 2020 (44)
SARS-CoV-2 RBD	HEK 293T	Produced in-house	Amanat *et al.* 2020 (44)
SARS-CoV-2 S2 subunit	HEK 293T	Sino Biological	
HCoV-OC43 S1 subunit	HEK 293T	Sino Biological	
HCoV-OC43 S2 subunit	Insect cells	Sino Biological	
HCoV-OC43 N protein	*Escherichia coli*	The Native Antigen Company	

Consortium details

ISARIC4C consortium:

J Kenneth Baillie, Malcolm G Semple, Peter JM Openshaw, Gail Carson, Beatrice Alex, Benjamin Bach, Wendy S Barclay, Debby Bogaert, Meera Chand, Graham S Cooke, Annemarie B Docherty, Jake Dunning, Ana da Silva Filipe, Tom Fletcher, Christopher A Green, Ewen M Harrison, Julian A Hiscox, Antonia Ying Wai Ho, Peter W Horby, Samreen Ijaz, Saye Khoo, Paul Klenerman, Andrew Law, Wei Shen Lim, Alexander J Mentzer, Laura Merson, Alison M Meynert, Mahdad Noursadeghi, Shona C Moore, Massimo Palmarini, William A Paxton, Georgios Pollakis, Nicholas Price, Andrew Rambaut, David L Robertson, Clark D Russell, Vanessa Sancho-Shimizu, Janet T Scott, Thushan de Silva, Louise Sigfrid, Tom Solomon, Shiranee Sriskandan, David Stuart, Charlotte Summers, Richard S Tedder, Emma C Thomson, Roger Thompson AA, Ryan S Thwaites, Lance CW Turtle, Maria Zambon, Hayley Hardwick, Chloe Donohue, Ruth Lyons, Fiona Griffiths, Wilna Oosthuyzen, Lisa Norman, Riinu Pius, Tom M Drake, Cameron J Fairfield, Stephen Knight, Kenneth A Mclean, Derek Murphy, Catherine A Shaw, Jo Dalton, James Lee, Daniel Plotkin, Michelle Girvan, Egle Saviciute, Stephanie Roberts, Janet Harrison, Laura Marsh, Marie Connor, Sophie Halpin, Clare Jackson, Carrol Gamble, Gary Leeming,

Andrew Law, Murray Wham, Sara Clohisey, Ross Hendry, James Scott-Brown, . William Greenhalf, Victoria Shaw, Sarah McDonald, Seán Keating, Katie A. Ahmed, Jane A Armstrong, Milton Ashworth, Innocent G Asiimwe, Siddharth Bakshi, Samantha L Barlow, Laura Booth, Benjamin Brennan, Katie Bullock, Benjamin WA Catterall, Jordan J Clark, Emily A Clarke, Sarah Cole, Louise Cooper, Helen Cox, Christopher Davis, Oslem Dincarslan, Chris Dunn, Philip Dyer, Angela Elliott, Anthony Evans, Lorna Finch, Lewis WS Fisher, Terry Foster, Isabel Garcia-Dorival, Willliam Greenhalf, Philip Gunning, Catherine Hartley, Antonia Ho, Rebecca L Jensen, Christopher B Jones, Trevor R Jones, Shadia Khandaker, Katharine King, Robyn T. Kiy, Chrysa Koukorava, Annette Lake, Suzannah Lant, Diane Latawiec, L Lavelle-Langham, Daniella Lefteri, Lauren Lett, Lucia A Livoti, Maria Mancini, Sarah McDonald, Laurence McEvoy, John McLauchlan, Soeren Metelmann, Nahida S Miah, Joanna Middleton, Joyce Mitchell, Shona C Moore, Ellen G Murphy, Rebekah Penrice- Randal, Jack Pilgrim, Tessa Prince, Will Reynolds, P. Matthew Ridley, Debby Sales, Victoria E Shaw, Rebecca K Shears, Benjamin Small, Krishanthi S Subramaniam, Agnieska Szemiel, Aislynn Taggart, Jolanta Tanianis-Hughes, Jordan Thomas, Erwan Trochu, Libby van Tonder, Eve Wilcock, J. Eunice Zhang, Kayode Adeniji, Daniel Agranoff, Ken Agwuh, Dhiraj Ail, Ana Alegria, Brian Angus, Abdul Ashish, Dougal Atkinson, Shahedal Bari, Gavin Barlow, Stella Barnass, Nicholas Barrett, Christopher Bassford, David Baxter, Michael Beadsworth, Jolanta Bernatoniene, John Berridge, Nicola Best, Pieter Bothma, David Brealey, Robin Brittain- Long, Naomi Bulteel, Tom Burden, Andrew Burtenshaw, Vikki Caruth, David Chadwick, Duncan Chambler, Nigel Chee, Jenny Child, Srikanth Chukkambotla, Tom Clark, Paul Collini, Catherine Cosgrove, Jason Cupitt, Maria-Teresa Cutino-Moguel, Paul Dark, Chris Dawson, Samir Dervisevic, Phil Donnison, Sam Douthwaite, Ingrid DuRand, Ahilanadan Dushianthan, Tristan Dyer, Cariad Evans, Chi Eziefula, Chrisopher Fegan, Adam Finn, Duncan Fullerton, Sanjeev Garg, Sanjeev Garg, Atul Garg, Effrossyni Gkrania-Klotsas, Jo Godden, Arthur Goldsmith, Clive Graham, Elaine Hardy, Stuart Hartshorn, Daniel Harvey, Peter Havalda, Daniel B Hawcutt, Maria Hobrok, Luke Hodgson, Anil Hormis, Michael Jacobs, Susan Jain, Paul Jennings, Agilan Kaliappan, Vidya Kasipandian, Stephen Kegg, Michael Kelsey, Jason Kendall, Caroline Kerrison, Ian Kerslake, Oliver Koch, Gouri Koduri, George Koshy, Shondipon Laha, Steven Laird, Susan

Larkin, Tamas Leiner, Patrick Lillie, James Limb, Vanessa Linnett, Jeff Little, Michael MacMahon, Emily MacNaughton, Ravish Mankregod, Huw Masson, Elijah Matovu, Katherine McCullough, Ruth McEwen, Manjula Meda, Gary Mills, Jane Minton, Mariyam Mirfenderesky, Kavya Mohandas, Quen Mok, James Moon, Elinoor Moore, Patrick Morgan, Craig Morris, Katherine Mortimore, Samuel Moses, Mbiye Mpenge, Rohinton Mulla, Michael Murphy, Megan Nagel, Thapas Nagarajan, Mark Nelson, Igor Otahal, Mark Pais, Selva Panchatsharam, Hassan Paraiso, Brij Patel, Natalie Pattison, Justin Pepperell, Mark Peters, Mandeep Phull, Stefania Pintus, Jagtur Singh Pooni, Frank Post, David Price, Rachel Prout, Nikolas Rae, Henrik Reschreiter, Tim Reynolds, Neil Richardson, Mark Roberts, Devender Roberts, Alistair Rose, Guy Rousseau, Brendan Ryan, Taranprit Saluja, Aarti Shah, Prad Shanmuga, Anil Sharma, Anna Shawcross, Jeremy Sizer, Manu Shankar-Hari, Richard Smith, Catherine Snelson, Nick Spittle, Nikki Staines, Tom Stambach, Richard Stewart, Pradeep Subudhi, Tamas Szakmany, Kate Tatham, Jo Thomas, Chris Thompson, Robert Thompson, Ascanio Tridente, Darell Tupper-Carey, Mary Twagira, Andrew Ustianowski, Nick Vallotton, Lisa Vincent-Smith, Shico Visuvanathan, Alan Vuylsteke, Sam Waddy, Rachel Wake, Andrew Walden, Ingeborg Welters, Tony Whitehouse, Paul Whittaker, Ashley Whittington, Meme Wijesinghe, Martin Williams, Lawrence Wilson, Sarah Wilson, Stephen Winchester, Martin Wiselka,

Adam Wolverson, Daniel G Wooton, Andrew Workman, Bryan Yates, Peter Young

OPTIC consortium:
Martyna Borak, Stavros Dimitriadis, Thomas Fordwoh, Bryn Horsington, Sile Johnson, Jordan Morrow, Yolanda Warren, Charlie Wells

SNBTS consortium:
Carol Imlach Carol McInally, Lisa M Jarvis, Marc Turner
